# Thallium(I) treatment induces nucleolar stress to stop protein synthesis and cell growth

**DOI:** 10.1038/s41598-019-43413-1

**Published:** 2019-05-06

**Authors:** Yi-Ting Chou, Kai-Yin Lo

**Affiliations:** Department of Agricultural Chemistry, National Taiwan University, Taipei, Taiwan

**Keywords:** Nucleolus, Ribosome

## Abstract

Thallium is considered as an emergent contaminant owing to its potential use in the superconductor alloys. The monovalent thallium, Tl(I), is highly toxic to the animals as it can affect numerous metabolic processes. Here we observed that Tl(I) decreased protein synthesis and phosphorylated eukaryotic initiation factor 2α. Although Tl(I) has been shown to interact with the sulfhydryl groups of proteins and cause the accumulation of reactive oxygen species, it did not activate endoplasmic reticulum stress. Notably, the level of 60S ribosomal subunit showed significant under-accumulation after the Tl(I) treatment. Given that Tl(I) shares similarities with potassium in terms of the ionic charge and atomic radius, we proposed that Tl(I) occupies certain K^+^-binding sites and inactivates the ribosomal function. However, we observed neither activation of ribophagy nor acceleration of the proteasomal degradation of 60S subunits. On the contrary, the ribosome synthesis pathway was severely blocked, i.e., the impairment of rRNA processing, deformed nucleoli, and accumulation of 60S subunits in the nucleus were observed. Although p53 remained inactivated, the decreased c-Myc and increased p21 levels indicated the activation of nucleolar stress. Therefore, we proposed that Tl(I) interfered the ribosome synthesis, thus resulting in cell growth inhibition and lethality.

## Introduction

Thallium is a natural source of trace element in the earth’s crust, with the concentration usually ranging from 0.3 mg/kg to 0.6 mg/kg. Thallium appears as monovalent Tl(I) and trivalent Tl(III) compounds in the environment. The inorganic Tl(I) is more stable than Tl(III) in solutions, but Tl(III) is more stable in its organic form^1–3^. Thallium is considered as an emergent contaminant because of its potential use in the superconductor alloys. The demand of thallium from the industries continually increases, and its contamination is becoming an important issue^4^.

Thallium is considered as a highly toxic element^5^. People exposed to high concentrations of thallium were shown to develop defects in the nervous system, lung, heart, liver, and kidneys or even death^3,6^. The chronic toxicity of thallium includes anorexia, pain in the abdomen, alopecia, and blindness^6,7^. Although thallium is a highly toxic element, the mechanism of its toxicity has been studied to a much lesser degree. Thallium binds to the sulfhydryl group of proteins and mitochondrial membranes, inhibiting various physiological functions, enzymatic reactions, and respiration.^8^. Thallium treatment also shows defects in DNA synthesis, energy generation, protein synthesis, and cell cycle progression^9^ in the mammalian cell lines, including PC12 cells^10,11^, rat hepatocytes^12,13^, and human T-lymphoblastoid Jurkat cell line^14^.

As Tl(I) and potassium ion (K^+^) possess similar ionic radii and are both monovalent, thallium is easily absorbed and affects the metabolic process of potassium and other cation-dependent reactions. Ions are important for the structural stability of the ribosomal subunits^15^. A total of 116 magnesium ions and 88 monovalent cations, including sodium and potassium, are found in the large ribosomal subunit from *Haloarcula marismortui*^15^. If the existing ions were replaced by other kinds of ions, the functions of ribosomes would be influenced. For example, although the total substitution of Ca^2+^ for Mg^2+^ resulted in lower activity and conformational change of the 50S ribosomal subunits from *E*. *coli*, the replacement of Sr^2+^ or Ba^2+^ completely inactivated the 50S subunits^16^. Studies have shown that the administration of Tl(I) to mice resulted in the reduced protein synthesis and disaggregation of liver polyribosome^17^. When incubating the isolated ribosomal subunits with a low concentration of Tl(I), Tl(I) could replace potassium in terms of certain partial functions. However, the high concentration of Tl(I) inactivates translation, particular destabilizing the 60S subunits^17,18^. Under this situation, the structure of the 60S ribosomal subunit was altered using chymotrypsin as a molecular probe^17^.

In this study, we observed that Tl(I) also affects the 60S ribosome synthesis pathway. The blockage of the 60S subunit synthesis, but not the degradation of abnormal 60S, primarily causes the under-accumulation of the large subunits *in vivo*. The ribosomes are composed of two subunits, a small subunit and a large subunit, which are the 40S and 60S subunits in the eukaryotic cells, respectively. The 40S and 60S subunits are assembled from a large primary rRNA 47S transcribed by RNA polymerase I. The processing of this rRNA yields the 5.8S, 18S, and 28S rRNA. The 5S rRNA is separately transcribed by RNA polymerase III (See reviews^19–22^. The 5S, 5.8S, and 28S rRNAs assemble with 46 ribosomal proteins to constitute the 60S subunit, whereas the 18S rRNA and 33 ribosomal proteins constitute the 40S subunit^23–25^. Ribosome biogenesis is a complex process requiring up to 200 transacting factors. It is essential for cell growth and proliferation. The Tl(I) treatment significantly reduced the 60S ribosomal subunits in a dose-dependent manner. The application of Tl(I) altered rRNA processing, changed the localization and abundance of important nucleolar proteins, and blocked the export process of pre-ribosomes. The impairment of ribosome synthesis induced nucleolar stress, resulting in the cell cycle arrest and apoptosis.

## Results

### Tl(I) inhibited cell growth

**Figure 1:**
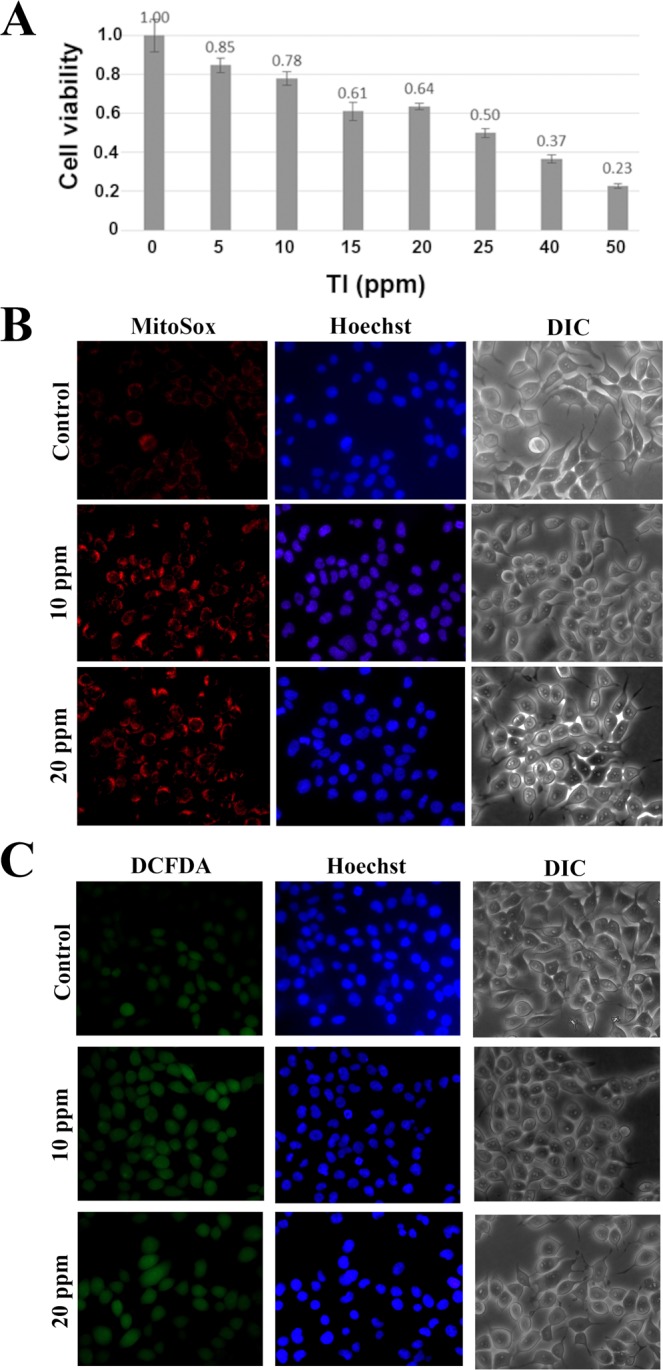
Tl(I) treatment reduced the cell numbers and induced oxidative stress of mammalian cells. (**A**) HEK293T, human embryonic kidney cells, were exposed to different concentrations of thallium for 24 h and then the viability was examined by MTT assay. ROS production was detected by MitoSOX Red (**B**) and DCFDA staining (**C**). The representative fluorescence images showed the ROS signals were enhanced under Tl(I) treatments compared to control. Scale bar, 20 μm.

We wanted to focus on the primary blockage of the physiological functions by thallium toxicity. Therefore, we checked the cell lethality at different concentrations of Tl(I) to determine the dosage for further study. After treatment of the HEK293 cell line with Tl(I) for 24 h, we observed that the cells showed 85%, 78%, and 64% viability at Tl(I) concentrations of 5, 10, and 20 ppm, respectively (Fig. 1A).

The toxicity from metals usually results in oxidative stress, which originates from the imbalance of ROS production and the ability of cells to scavenge such molecules. Although the ROS are important for the regulation of various physiological functions, their excess production damages proteins, nucleic acids, membrane and causes the progression of various diseases, i.e., aging and inflammation.We evaluated the oxidative stress stimulated by Tl(I) and observed the elevated production of ROS at Tl(I) concentrations of 10 and 20 ppm (Fig. 1B,C).

**Figure 2:**
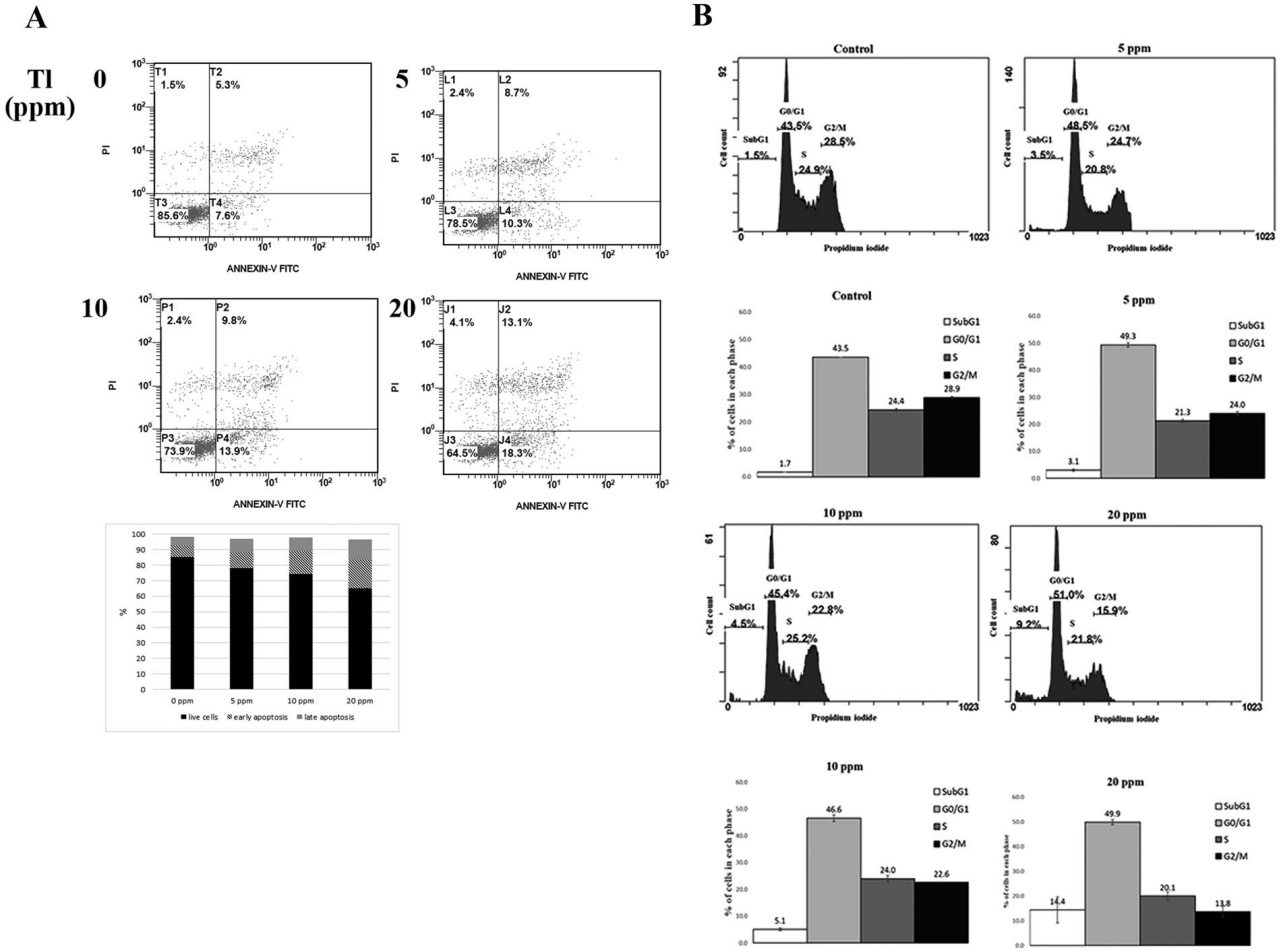
Thallium(I) triggered apoptosis and cell cycle arrest. (**A**) Detection of apoptosis by Annexing V and propidium iodide staining using flow cytometry in HEK293T. Representative scatter plots of Annexing V and propidium iodide staining of control or treatments of Tl(I) after 24 hours. The quantitative results of apoptotic cells were shown in the graph. (**B**) The cell cycle analyses were performed by flow cytometry using propidium iodide to stain DNA. The HEK293T cells were exposed to different concentrations of Tl(I) for 24 hours. The means from three biological repeats (n = 3) are displayed.

The cells were further analyzed if the apoptosis pathway was activated. The cells were treated with Tl(I) and stained with Annexin V and PI. The viable cells decreased (region 3, Fig. 2A), whereas the apoptotic cells increased (region 2 and 4, Fig. 2A) in response to the increasing Tl(I) concentrations. The cell cycle was also analyzed in the cells treated with various concentrations of Tl(I). The cells at G2/M decreased, and those at the sub-G1 phase elevated under the application of Tl(I) (Fig. 2B). Therefore, under the range of applied concentrations, Tl(I) induced apoptosis and blocked the cell cycle progression.

### Tl(I) decreased protein synthesis

**Figure 3:**
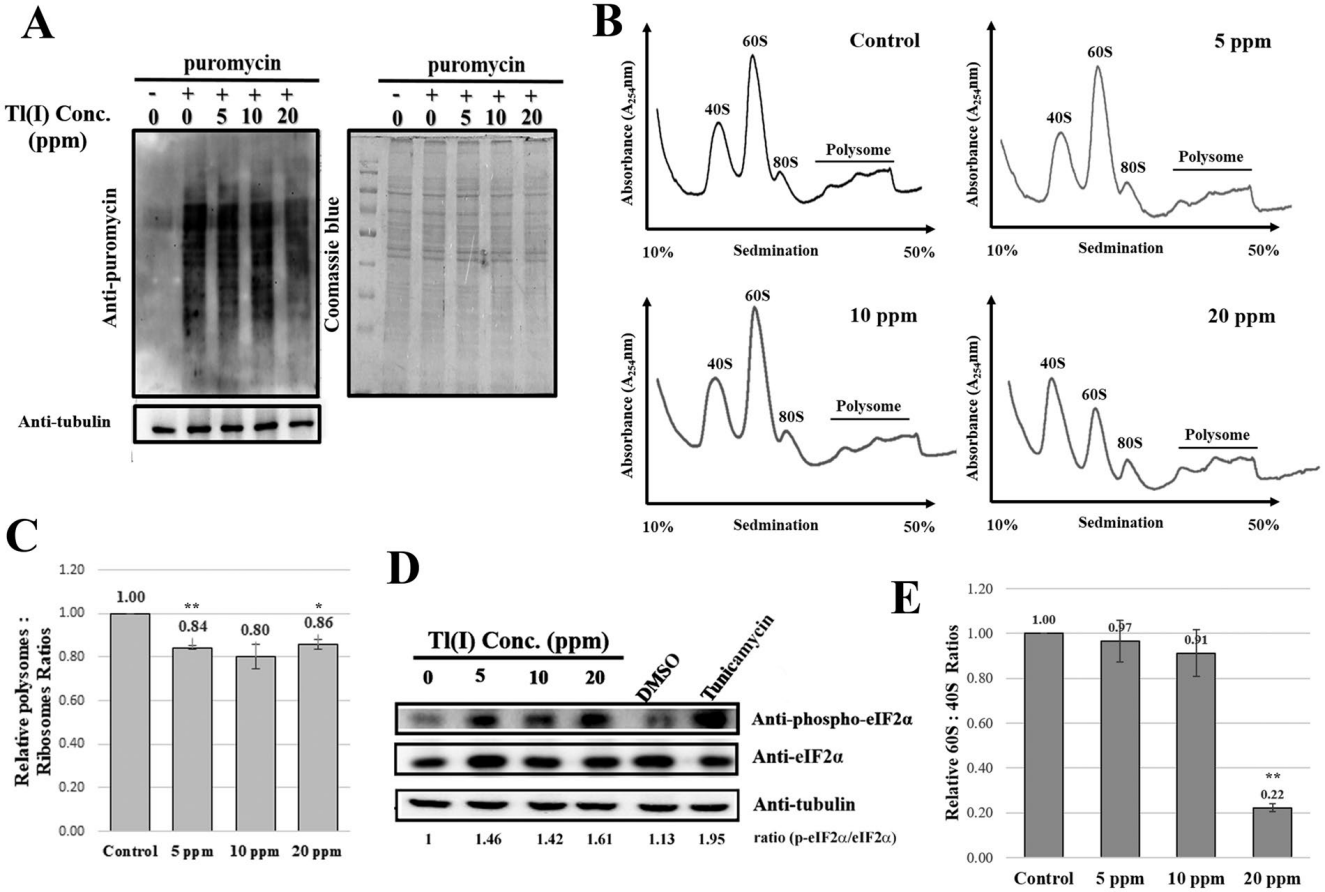
Thallium reduced global translation. (**A**) Protein synthesis was measured by SUnSET. Production of newly synthesized proteins labeled by puromycin was detected by anti-puromycin antibody. (**B**) After HEK293T cells were treated with different concentrations of Tl(I) for 24 hours, cells were harvest and lysed. The lysates were fractioned through 10% to 50% sucrose gradients. And the profiles were recorded using A_254_. (**C**) The resulting polysomes: ribosomes ratios were quantitated for control versus treatments as noted in the polyribosome profile analyses. (**D**) Phosphorylated eukaryotic translation initiation factor 2α (p-eIF2α) was detected by immunoblotting. Tunicamycin is a positive control of ER stress. (**E**) The calculated 60S/40S ratio from the profiles in 3B. The means from two biological repeats (n = 2) are displayed. Error bars represent SD. Student’s t tests relative to the control were performed. *p < 0.05, **p < 0.01. Uncropped gel images are shown in Supplementary Fig. S3.

To further dissect the effects of Tl(I) on the cells, the protein synthesis was analyzed with SUnSET^26^, a nonradioactive method for monitoring such process. Puromycin, a tRNA analog, can enter the tRNA-binding site of ribosome and conjugate with the peptides under synthesis. The puromycin-labeled nascent peptides could be detected by the anti-puromycin antibody. The level of nascent protein synthesis decreased with the increasing amount of Tl(I) (Fig. 3A). The polysome profiles were also used to assess the protein translation conditions. The amounts of translating ribosomes (Fig. 3B,C. polysome) decreased when Tl(I) was present in the culture. The polysome peaks indicate the amount of translating ribosomes bound on the mRNAs, showing the translation capacity. This observation supports the deficient synthesis of nascent proteins.

Stress usually inactivates global translation from the phosphorylation of eIF2α, preventing this subunit to join translation. This result was also observed when the cells were exposed to Tl(I) (Fig. 3D). Tl(I) has been shown to interact with the thiol groups of proteins and cause protein inactivation^8^. The accumulation of denatured proteins may activate the ER stress, which induces the phosphorylation of eIF2α and stops translation^27^. Tl(I) also induced the ROS production (Fig. 1B,C), which has been linked to ER stress. We examined whether Tl(I) decreased the protein synthesis through this route. However, none of the ER stress markers, including XBP1 (X-box binding protein 1), Bip, and CHOP (C/EBP homologous protein), was activated under Tl(I) treatment (data not shown).

### Tl(I) decreased the levels of 60S ribosomal subunits

From the polysome profiles, under-accumulation of the 60S ribosomal subunits was observed after Tl(I) exposure (Fig. 3B,E). In the normal cells, the 40S and 60S subunits should be in equivalent ratio. As the 60S ribosome features higher molecular weight than the 40S subunit, it exhibited stronger absorbance at the examined wavelength. The peak area was about 1.5- to 2-fold larger than that of the 40S subunit. Under the increasing amount of Tl(I), the 60S subunit decreased in size. At 20 ppm, the 60S peak was lower than that of the 40S peak (Fig. 3B).

**Figure 4:**
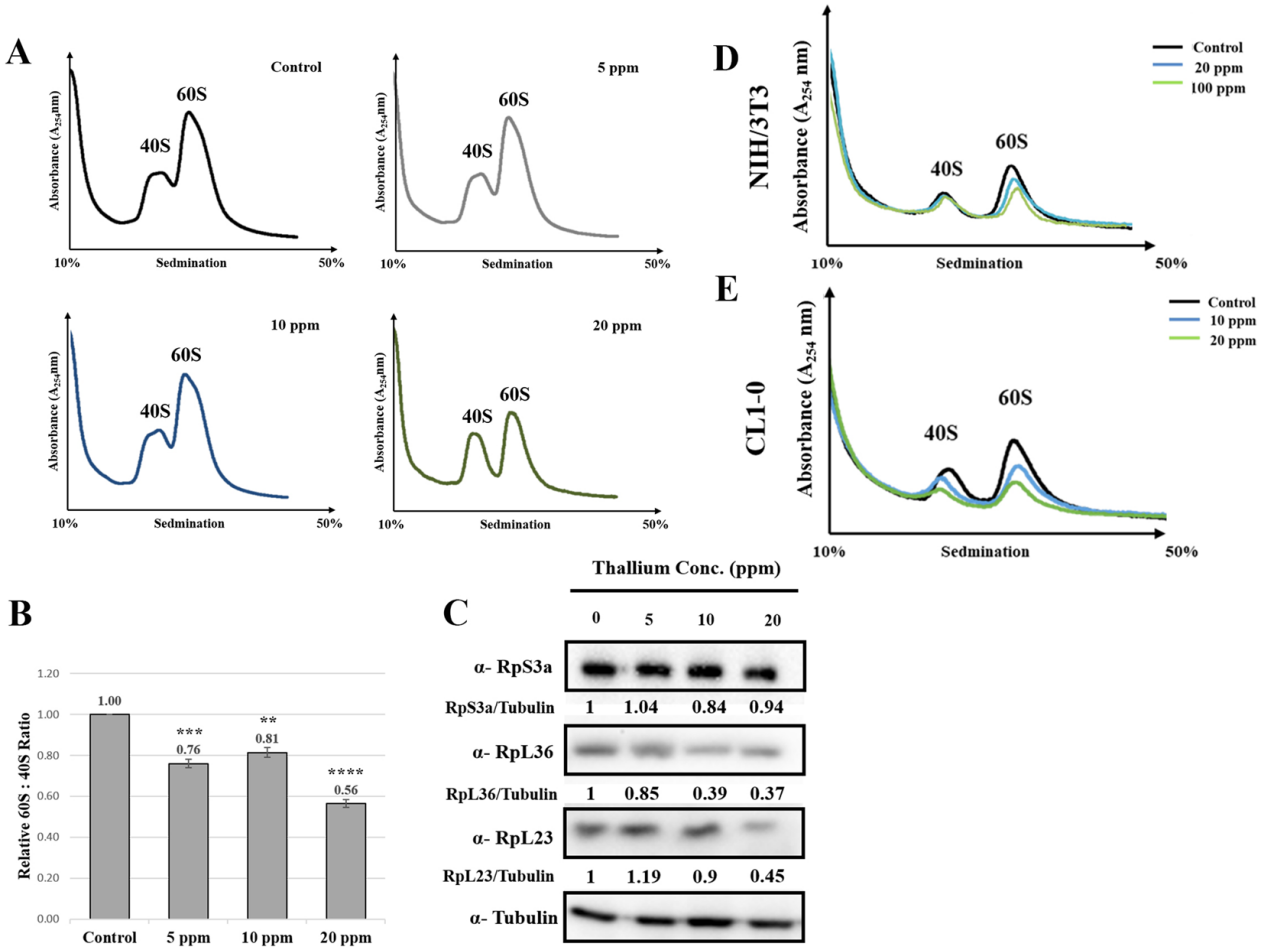
Thallium impacted the levels of 60S ribosomal subunits. (**A**) HEK293T was exposed to Tl(I) for 24 hours. The cells were lysed in 0.25 mM Mg^2+^ to dissociate the 40S and 60S ribosomal subunits. The resulting ribosomal profiles were analyzed by sucrose gradient sedimentation. (**B**) The resulting 60S:40S ratios were quantitated for control versus treatments as noted in the monosome profile analyses. The means from two biological repeats (n = 2) are displayed. Student’s t tests relative to the control were performed. **p < 0.01, ***p < 0.005, ****p < 0.001 (**C**) Ribosomal proteins of large subunit (i.e. RpL23, RpL36) and small subunit (i.e. RpS3a) were detected by immunoblotting. (**D**,**E**) NIH/3T3 (**D**) and CL1-0 (**E**) cells were treated with different amount of Tl(I) for 24 hours. Cells were harvested and lysed in the buffer with 0.25 mM Mg^2+^. Cell lysates were fractioned through 10% to 50% sucrose gradients and the profiles were recorded using A_254_. Uncropped gel images are shown in Supplementary Fig. S4.

To magnify the changes in the ribosomal subunits, the cells were disrupted in the low-Mg buffer and fractioned through sucrose density gradient. Under this situation, the 40S and 60S subunits dissociated and appeared as two separate peaks. Consistently, the 60S subunit decreased under exposure to high concentrations of Tl(I) (Fig. 4A,B). Similarly, the levels of ribosomal proteins of large subunits decreased (RpL23 and RpL36). However, the levels of ribosomal proteins of small subunits remained unchanged (RpS3a) (Fig. 4C).

We also checked the levels of ribosomal subunits in NIH/3T3, mouse embryonic fibroblast cells, and CL1-0, human lung cancer cells. For a fair comparison, we also treated cells at different Tl(I) concentrations to cause comparable reduction of the cell number similar in HEK293T cells. CL1-0 showed 80% and 60% cell growth at 10 and 20 ppm Tl(I), respectively. Concentrations were required to be elevated to 20 and 100 ppm resulted in similar growth reduction in NIH3T3 (data not shown). Consistently, the 60S subunit was also reduced under Tl(I) treatment (Fig. 4D,E). In the CL1-0 cells, the levels of 40S subunit also dropped under the treatments, but the reduction was not as significant as that of the 60S subunit (Fig. 4E).

### Reduction of the 60S ribosomal subunits was not from degradation of mature ribosomes but from underaccumulation of nascent subunits

**Figure 5:**
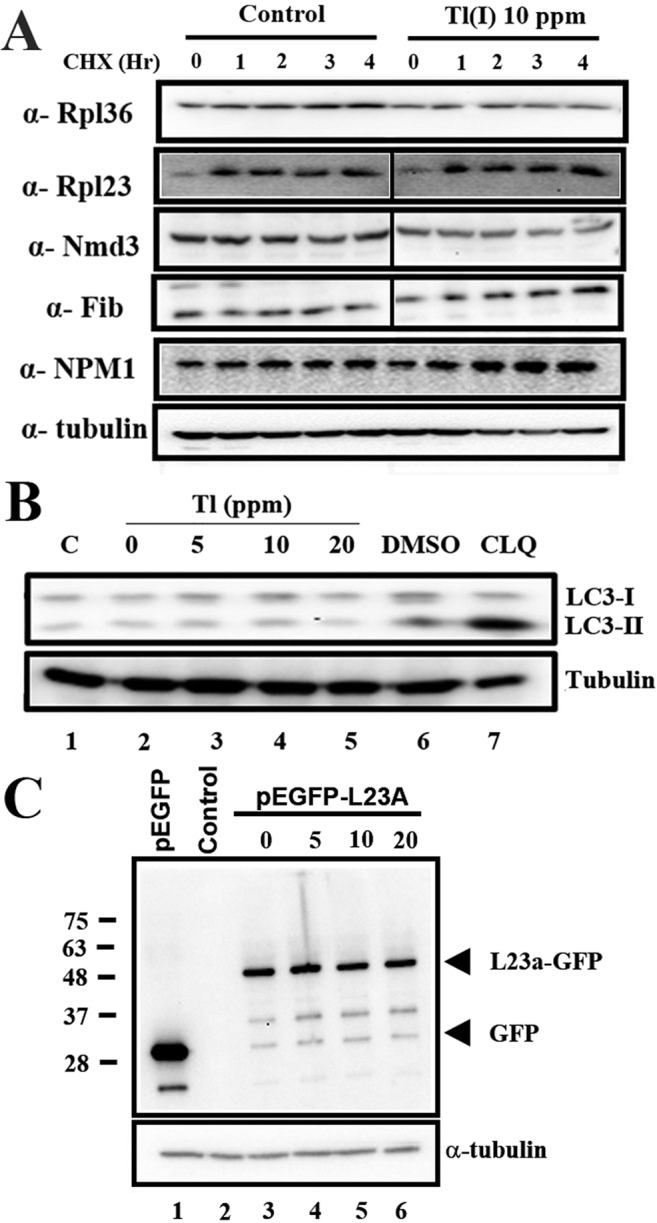
60S ribosomes were not decreased through proteasome or ribophagy pathway. (**A**) HEK293T cells were treated with 0.1 mg/ml cycloheximide (CHX) for the indicated time course. The protein levels of RpL36, RpL23, Nmd3, fibrillarin, NPM1 and tubulin were detected by Western blotting. (**B**) Western blot of the autophagy marker LC3. Chloroquine (CLQ) is the positive control of autophagy. (**C**) The HEK293T cells expressing RpL23a-GFP were treated with different concentrations of Tl(I). Degradation of GFP-tagged proteins was analyzed by anti-GFP antibody. Uncropped gel images are shown in Supplementary Fig. S5.

The lower 60S peak possibly originated from the specific degradation or blockage of biogenesis of the large ribosomal subunits. As Tl(I) and potassium are remarkably similar, Tl(I) may replace potassium and inactivate the ribosomal functions^17,18^. Therefore, we first examined if Tl(I) reduced the level of 60S ribosome from the elimination of inactive ribosomal subunits. CHX was applied to stop protein synthesis, and the protein levels of ribosomal proteins (i.e., RpL36 and RpLl23) and transacting factors (i.e., Nmd3, fibrillarin, and NPM1) were tracked at different time points for evaluation of stability. The degradation rate did not accelerate under exposure to Tl(I) (Fig. 5A).

Ribosome could be degraded through autophagy, specifically ribophagy, under starvation or elimination of the dysfunctional subunits. Microtubule-associated protein LC3 is wildly used as an autophagy marker. During autophagy, LC3-I is cleaved at the carboxy terminus and yields LC3-II for association with autophagosomes. Therefore, the elevated level of cleaved LC3 form is used as an indicator of autophagy^28^. While the autophagy activator, chloroquine (CLQ), increased the level of LC3-II, Tl(I) treatment did not result any changes (Fig. 5B). In addition, RpL23a-GFP was used as a reporter for ribophagy. If the ribosomes were degraded through the autophagy route, an increase in GFP would be observed given that this molecule is more resistant to degradation^29^. Consistent with the results above, the GFP level did not increase under Tl(I) treatments (Fig. 5C). Hence, the lower 60S levels, instead of being caused by degradation, resulted from other events (Fig. 5).

**Figure 6:**
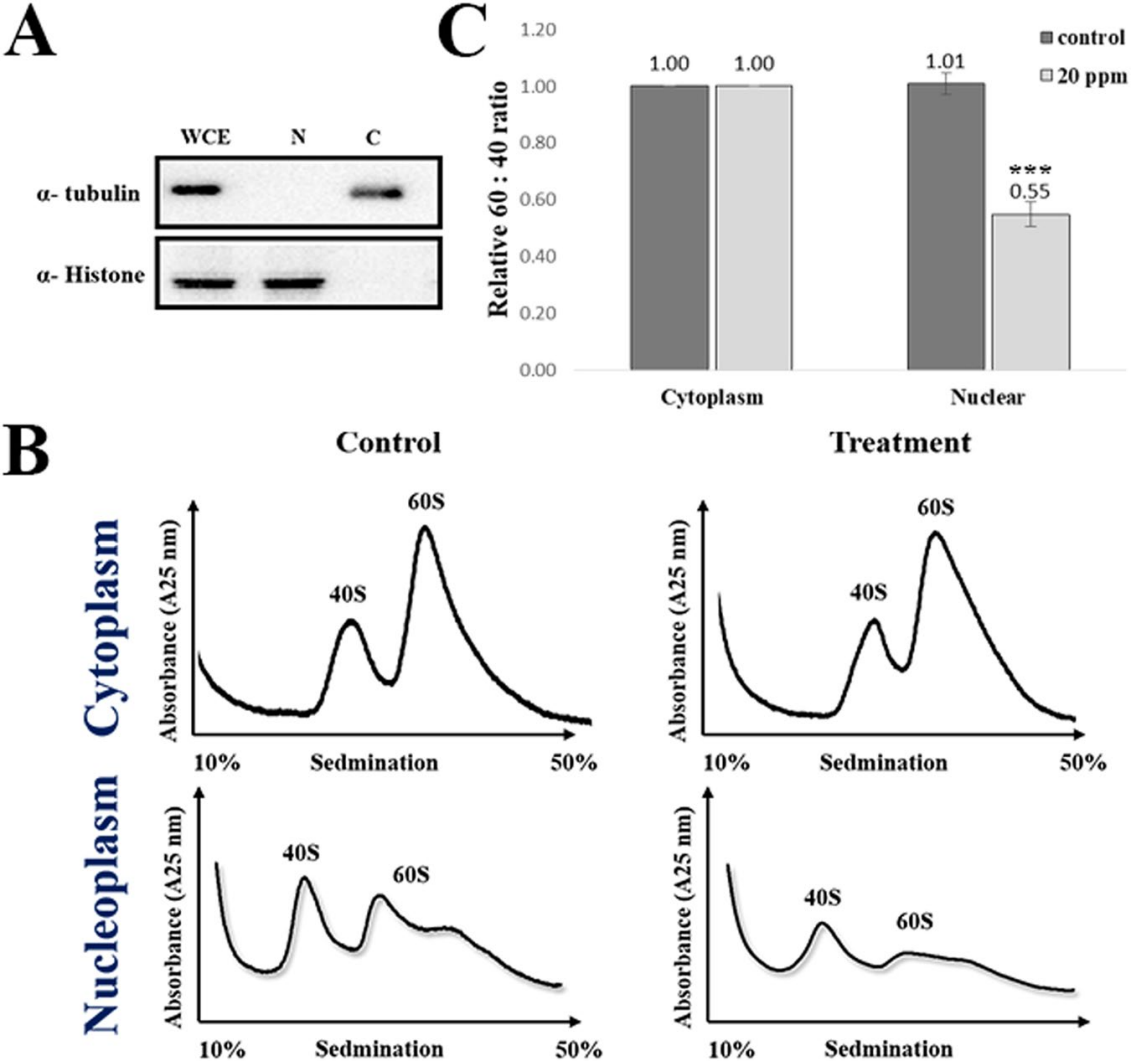
The level of pre-60S was reduced under exposure of Tl(I). (**A**) α-Tubulin and histone were used as the loading controls for cytoplasmic and nuclear extracts, respectively, for cell fractionation. WCE, whole cell extracts. (**B**) HEK293T cells were treated with (20 ppm) or without (0 ppm) Tl(I) for 24 hours. The cells were lysed in the buffer with 0.25 mM Mg^2+^−0.1% NP-40 and centrifuged. The supernatant was cytoplasmic fraction. And the nuclear pellets were resuspended in 0.5 ml of sonication buffer (25 mM Tris pH 7.5, 100 mM KCl, 2 mM EDTA, 0.05% NP-40), and sonicated on ice. Extracts were fractioned through 10% to 50% sucrose gradients and the profiles were recorded using A_254_. (**C**) The resulting 60S:40S ratios were quantitated for control versus treatments as noted in the monosome profile analyses. The means from two biological repeats (n = 2) are displayed. Student’s t tests relative to the control were performed. ***p < 0.005. Uncropped gel images are shown in Supplementary Fig. S6.

To further dissect if the decrease was caused by the cytoplasmic or nuclear pool of the 60S subunit, we fractioned the cells (Fig. 6A) and analyzed the levels of ribosomal subunits through the sucrose gradients (Fig. 6B). Interestingly, the levels of ribosomes and the ratio between the 40S and 60S subunits from the cytosol remained constant between the control and 20 ppm-treated cells. However, the 60S level in nucleoplasm dropped significantly after the Tl(I) treatment (Fig. 6B,C). This finding suggests that Tl(I) affects the accumulation of pre-ribosomes but not the mature ribosomes.

**Figure 7:**
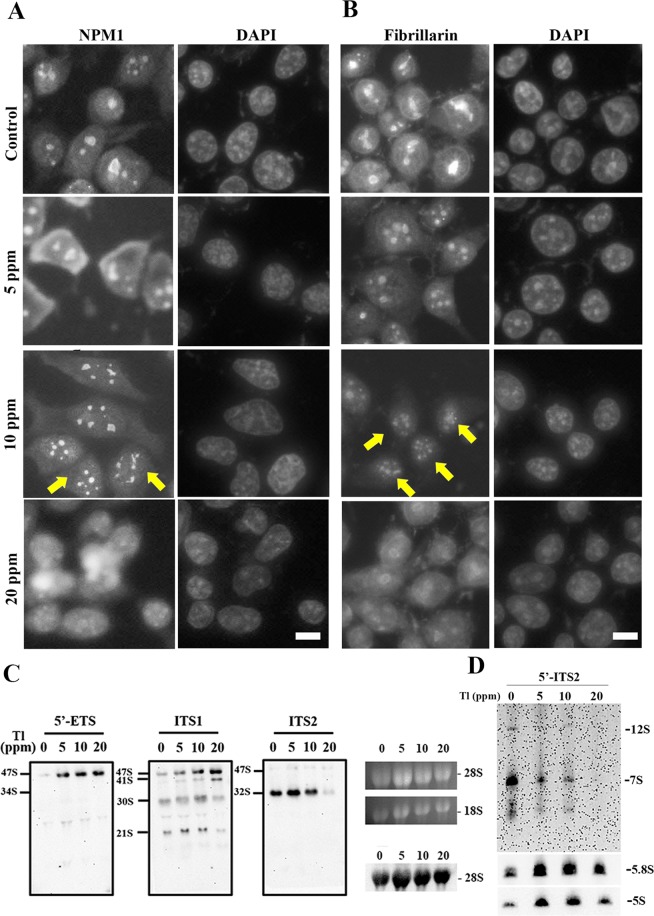
Tl(I) induced mislocalization of nucleolar proteins and impaired rRNA processing. (**A**,**B**) Immunofluorescence staining of NPM1 (**A**) and fibrillarin (**B**) in HEK293T cells that have been treated with different concentrations of thallium. DNA was stained with DAPI. Cells with nucleolar fragmentation and necklace formation were indicated by yellow arrows. Scale bars, 10 μm. (**C**,**D**) Analysis of pre-rRNA processing. Total RNAs were extracted from cells and analyzed by Northern blot with probes for 5′-ETS (probe 1), ITS1 (probe 2), ITS2 (probe 3), 5′-ITS2 (probe 4), 28S, 5.8S and 5S. The signals of 28S, 5.8S and 5S in the Northern blotting, and 28S and 18S rRNAs stained with EtBr were shown as the loading controls. Pre-rRNA processing pathway and uncropped gel images are shown in Supplementary Fig. S7.

To further analyze if Tl(I) altered the ribosome biogenesis, the morphology of nucleolus, the compartment where most ribosome synthesis occurs, was examined. NPM1 (B23/nucleophosmin) is a multifunctional nuclear acidic chaperone required in most stages of ribosome biogenesis^30^. Fibrillarin is an essential rRNA methyltransferase localized in the nucleolus, marked for active RNA polymerase I^31^. In the controls, both NPM1 and fibrillarin localized in the nucleolus (Fig. 7A,B, respectively). At 5 ppm, NPM1 maintained nucleolar enrichment but partially lost the nuclear signal. At 10 ppm and 20 ppm, NPM1 was shown as less condensed structures resembling necklace (indicated by yellow arrows) and disrupted nucleolar signals, respectively (Fig. 7A). Fibrillarin showed normal spherical shape of nucleolus but stronger cytoplasmic signals at 5 ppm. When Tl(I) increased to 10 ppm, nucleolar fragmentation and necklace formation exhibited (Fig. 7B indicated by yellow arrows). At 20 ppm, most fibrillarin was lost from the nucleolus (Fig. 7B). Tl(I) treatment induced abnormalities in nucleolar structure and mislocalizations of critical nucleolar proteins. This implies impairment of ribosome synthesis.

### Tl(I) treatment impaired rRNA processing and ribosome biogenesis

To further investigate how thallium influences the ribosome synthesis pathway, we first examined the rRNA processing in cells treated with thallium. In human cells, 18S, 5.8S and 28S rRNAs are produced by RNA polymerase I on a single transcript. Following this event, a number of cleavage steps transpire to produce the final mature rRNAs (Fig. 7 Supplement). rRNA processing starts from the nucleolus and finishes in the cytoplasm. Nascent ribosomal subunits contain various pre-rRNAs, ribosomal proteins, and transacting factors. The compositions change dynamically and quickly during the synthesis steps. Northern blotting was performed to identify the rRNA processing steps influenced by thallium. The probes specific to the 5ʹ-external transcribed spacers (ETS), internal transcribed spacers (ITS) 1, ITS2, and 5ʹ-ITS2 regions were used to detect the pre-rRNAs (Supplementary Fig. S7).

Under the Tl(I) treatment, the 47S and 41S pre-rRNAs accumulated, whereas the downstream products, the 32S, 30S, 21S, 12S and 7S pre-rRNAs, decreased significantly (Fig. 7C,D). Therefore, rRNA processing was blocked at the very early stages by Tl(I).

**Figure 8:**
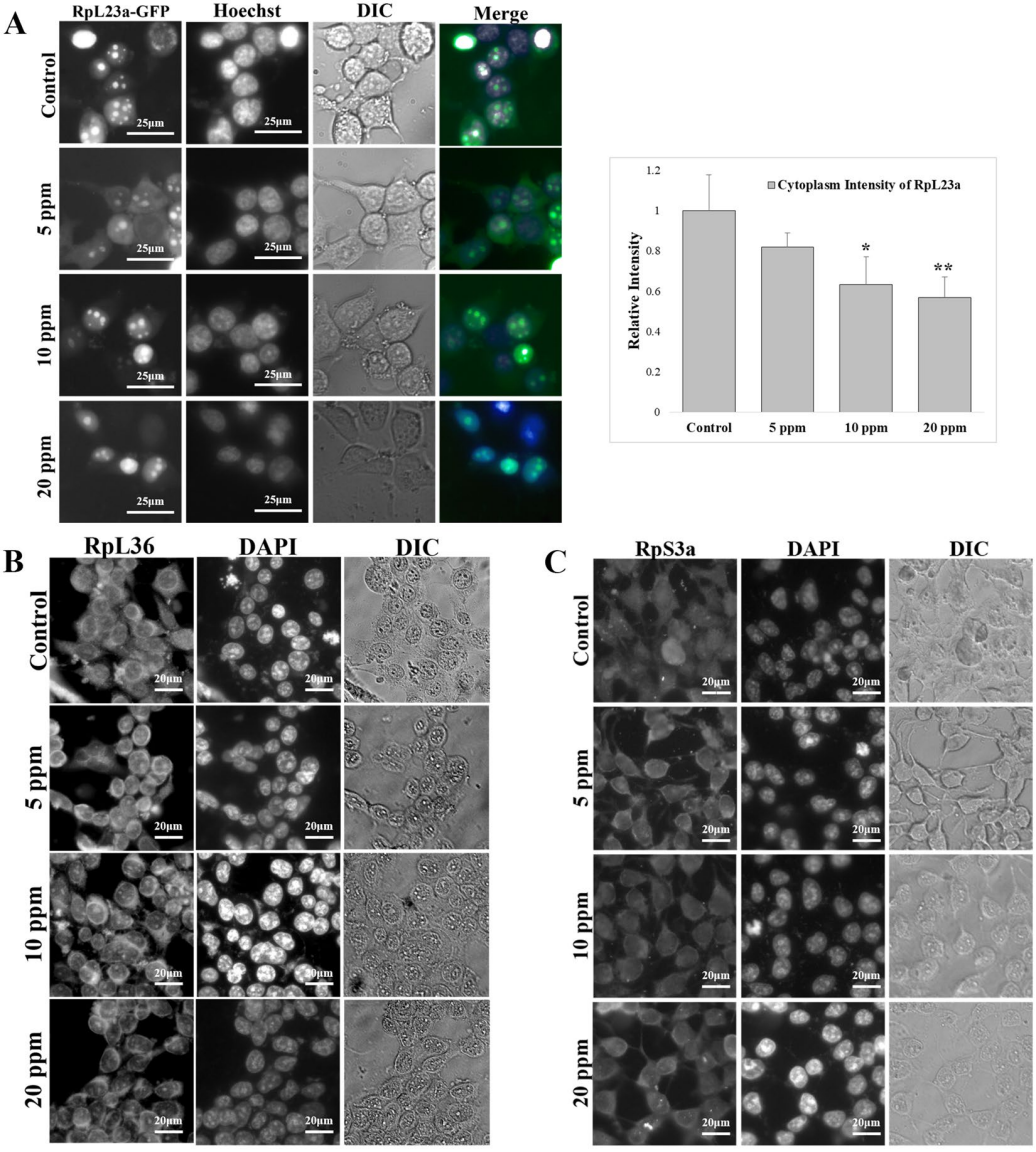
The localization of large ribosomal proteins were nuclear accumulation in treatment groups. (**A**) The HEK293T cells expressing RpL23a-GFP were treated with different concentrations of thallium. And the protein localization of RpL23a was imaged by fluorescent microscopy. Cell nuclei were stained with Hoechst. The cytoplasmic intensity of RpL23a was measured with Image J and shown in the bar graph (n = 20). Scale bars, 25 μm. Error bars represent SD. Student’s t tests relative to the control were performed. *p < 0.05, **p < 0.01. (**B**,**C**) Immunofluorescence staining of RpL36 (**B**) and RpS3a (**C**) with anti-RpL36 and anti-RpS3a. DNA was stained by DAPI. Scale bars, 20 μm.

We also tracked the cellular distribution of the ribosomal subunits. To track the 60S subunits, we used RpL23A-GFP and RpL36 as reporters. The cells transfected with RpL23A-GFP showed GFP signals in the cytoplasm, nucleoplasm, and nucleolus in the controls. Under Tl(I) exposure, the cytoplasmic signals were decreased (Fig. 8A). RpL36 was detected by immunofluorescence with the anti-RpL36 antibody. A stronger accumulation of RpL36 in the nucleolus was observed, whereas the cytoplasmic signal became weaker (Fig. 8B). By contrast, RpS3a showed no change under exposure to 20 ppm Tl(I) (Fig. 8C).

### Tl(I) affected numerous genes in the ribosome biogenesis pathway

**Figure 9:**
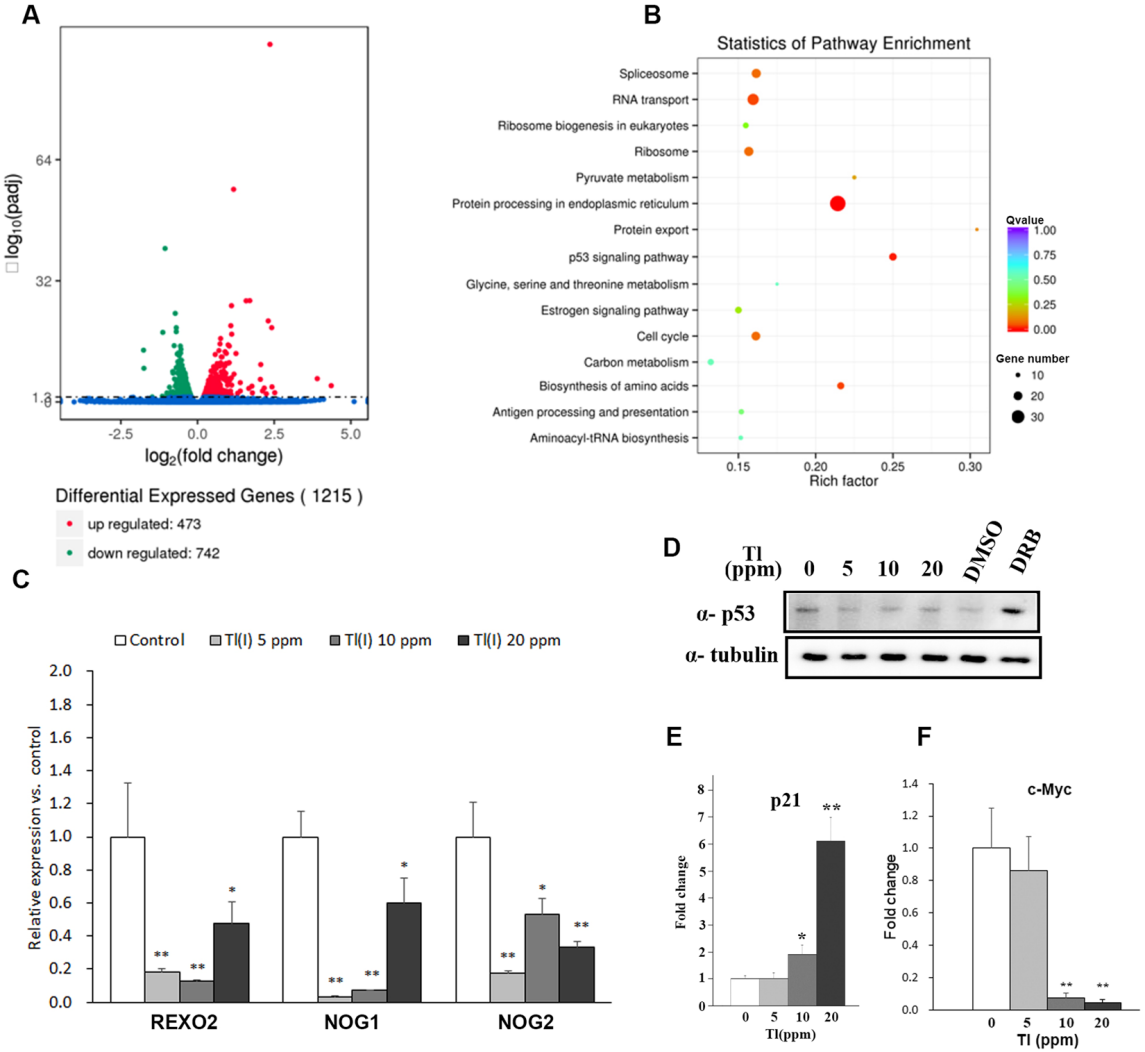
Tl(I) disturbed the gene expressions involved in ribosome synthesis pathway and induced p53-independent nucleolar stress. (**A**) Volcano plot showed the differentially expressed genes. (**B**) KEGG enrichment scattered plot. A graphical display way of KEGG enrichment analysis results. Top 15 most significant enriched pathways were chosen in KEGG scatter plot. Rich factor is the ratio of DEGs counts to this pathway in the annotated genes counts. If the Rich factor is bigger, it indicates higher degree of enrichment. Q-value is the adjusted p-value after multiple hypothesis testing, and its range is [0, 1]. When the Q-value is close to zero, this means the enrichment is more significant. (**C**) RT-qPCR results for quantification of gene expressions involved in ribosome biogenesis. Mean ± SD (n = 4). * indicate difference vs control at p < 0.1; ** indicate difference vs control at p < 0.05, ANOVA followed by LSD test. (**D**) Immunoblotting of p53. (**E**,**F**) RT – qPCR of c-Myc and p21. Mean ± SD (n = 4). Uncropped gel images are shown in Supplementary Fig. S9.

The transcriptome of Tl(I)-treated cells was analyzed using NGS technology (Illumina, San Diego, CA, USA), and the results were obtained in duplicate. The square of the Pearson correlation coefficients of the controls and 20 ppm Tl(I)-treated samples were 0.992 and 0.991, respectively. These values demonstrate the small differences in the gene expressions between the samples in the same experimental group. A total of 1215 genes showed statistical significance (q value < 0.05) and were differentially expressed in two groups. Among these, 473 genes were upregulated, and 742 genes were downregulated (Fig. 9A).

To analyze the changes in gene expressions, the databases, Gene Ontology and KEGG, were used. The major groups changed in 20 ppm Tl(I) treatment, showing changes in protein export (7 genes among 23 genes), biosynthesis of amino acids (16 genes among 74 genes), RNA transport (26 genes among 163 genes), spliceosome (21 genes among 130 genes), ribosomal proteins (21 genes among 134 genes), and ribosome biogenesis in eukaryotes (13 genes among 84 genes) (Fig. 9B). All these pathways are connected with the ribosome synthesis.

To further confirm that Tl(I) changed the ribosome synthesis pathway, several genes involved in the ribosome biogenesis were selected for qPCR analyses. RNA exonuclease 2 (REXO2) is a 3ʹ-to-5ʹ exonuclease^32^ and plays roles in DNA repair, replication, and recombination, and in 3ʹ-end processing of U4 and U5 snRNAs and 5S and 5.8S rRNAs^33^. Nucleolar GTP-binding protein 1 (NOG1) and NOG2 (also called NUG2) are GTPases and important for the ribosome biogenesis^34^. In the qPCR results, the mRNA levels of REXO2, NOG1, and NOG2 significantly reduced under 5, 10, and 20 ppm Tl(1) exposure (Fig. 9C).

### Tl(I) induced p53-independent nucleolar stress response

Impaired ribosome synthesis induces nucleolar stress response. Nucleolar stress may enhance the level of p53, resulting in the arrest of cell cycle or induction of apoptosis. To test this possibility, the transcription and protein level of p53 were examined with qPCR and Western blotting, respectively. However, p53 exhibited no elevation under Tl(I) treatment under either analyses (Fig. 9D; data not shown).

Nucleolar stress could be also activated via the p53-independent pathway^35^. In the NGS results, although the p53 signaling pathway was one of the most significantly enriched pathways after Tl(I) treatment (Fig. 9B), the expression level of p53 gene remained unchanged, consistent with the qPCR results. Instead, p21, a cyclin-dependent kinase inhibitor, was highly elevated in the NGS assay. Its accumulation also resulted in the cell cycle arrest and apoptosis. We confirmed this finding with qPCR and observed that p21 increased to about 6-fold at 20 ppm treatment (Fig. 9E). c-Myc is a master regulator, controlling the transcription of the three RNA polymerases. Excess ribosomal proteins from impaired ribosome biogenesis would reduce the level of c-Myc, leading to a decreased cell number in the S phase^36^. Consistently, the transcription level of c-Myc decreased pronouncedly at 10 and 20 ppm (Fig. 9F). The data above indicate that Tl(I) disturbed the ribosome synthesis and induced p53-independent nucleolar stress. This condition decreased the global protein synthesis and further arrested the cell cycle or triggered apoptosis.

## Discussion

### Tl(I) decreased the global translation from phosphorylation of eIF2α and blocked the 60S subunit biogenesis

The cellular 60S subunit levels decreased after Tl(I) treatment. As Tl(I) and potassium are very similar, Tl(I) may replace potassium and inactivate the ribosomal functions. Studies have shown that the incubation of isolated ribosome with Tl(I) inactivated ribosomal functions in an *in vitro* translation system^17,18^. In our study, we observed neither elimination of mature ribosomes through the proteasome nor the autophagy route.

The ribosome biogenesis, especially that of the large subunits, was impaired by Tl(I) in the nucleus/nucleolus. The ribosome biogenesis is a complicated process, involving more than 200 transacting factors. The biosynthesis of large subunits is more complicated than that of small subunits. The transacting factors include proteins with enzyme activity, such as ATPase, GTPase, and methyltransferase. One and five GTPases are shown to join the synthesis of the 40S and 60S ribosomal subunits, respectively. Three GTPases, Nug1, Nog2 (also called Nug2), and Lsg1, are K^+^-dependent and function in the biogenesis of large subunits^34^. One possibility is that Tl(I) competes with potassium for the binding sites of these enzymes and inactivates their functions. Nug1 is required for formation of peptidyl transferase center (PTC). The inactivation of Nug1 causes the blockage of processing at A2 in yeast, which is comparable to that at site 2 in humans^37–39^. Nog2 binds at the PTC site and is implicated in the 27SB to 7S processing^40,41^. Nog2 and Nog1 interact with multiple assembly factors and functional rRNA elements to remodel the pre-60S subunit. These GTPases are critical for the ITS2 removal and 60S export^42^. Lsg1 is a cytoplasmic protein that is required for releasing the essential export adapter Nmd3 from the 60S subunit and allows Nmd3 to return to the nucleus for another round of ribosome transportation^43,44^. In Tl(I)-treated cells, the accumulation of 47S and 41S rRNA, and under-accumulation of 30S, 32S, and 21S rRNA were observed. This finding is possibly due to the blockage of processing at site 2, potentially from the partial impairment of Nug1 and Nog2.

### Tl induced p53-independent nucleolar stress

Stress usually inactivates the global translation from phosphorylation of eIF2α. This was also observed in the cells exposed to Tl(I). Tl(I) has been shown to interact with the thiol groups of proteins and cause protein inactivation. The accumulation of denatured proteins may activate the ER stress, which induces the phosphorylation of eIF2α and stops translation. The elevation of ROS triggered by Tl(I) can result in a reduction–oxidation imbalance and aggravate the ER stress through reducing the efficiency of protein folding pathways^45^. We examined the potential activations of several ER stress markers. However, neither one was activated under Tl(I) treatment.

Nucleolar stress is characterized by abnormalities in nucleolar structure and function. This leads to activation of p53 or other stress signaling pathways and alterations in cell behavior, i.e. cell cycle arrest or apoptosis^35,46,47^. Under normal conditions, ribosomal proteins translated in the cytoplasm are rapidly transported to the nucleus for incorporating into the nascent ribosomal subunits. The disturbance of ribosome synthesis causes accumulation of free ribosomal proteins, resulting in stabilization of p53^48^. However, activation of p53 was not observed in our study.

Excess ribosomal proteins could also induce p53-independent nucleolar stress^35^. c-Myc is a master regulator controlling the transcription of the three RNA polymerases^49^. Excess ribosomal proteins bind both c-Myc and its mRNA, reducing the level of c-Myc and the number of cells in the S phase^36^. p21 is a primary inhibitor of cyclin-dependent kinases. Under the impairment of ribosome biogenesis, a complex of RpL3 and NPM1 binds to the p21 promoter and causes the upregulation of p21 expression^50^. These two are common factors involved in p53-independent nucleolar stress. The levels of c-Myc and p21 mRNAs decreased and increased, respectively, in response to the concentrations of Tl(I). Though we cannot exclude the possibility that reduction of c-Myc after Tl treatment is the first cause to impair ribosome synthesis from our data. Both situations activate nucleolar stress.

While the reduction of 60S subunits was not significant at 10 ppm (Figs 3B, 4A), the decrease of c-Myc mRNA was dramatic (Fig. 9F). The decrease of RpL36 (Fig. 4C), and abnormalities in nucleolar structure (Fig. 7A) and rRNA processing (Fig. 7C,D) were still observed at 10 ppm. Therefore, the cellular response might correlate to the Tl(I) concentration. In conclusion, the cells exposed to Tl(I) exhibited decreased protein synthesis and impaired ribosome biogenesis, further triggering blockage of cell cycle progression and apoptosis.

## Materials and Methods

### Cell culture and miscellaneous

Human embryonic kidney 293 (HEK293) T and CL1-0 cell lines were cultured in Dulbecco’s Modified Eagle Medium (DMEM) (Gibco) with 5% fetal bovine serum, and NIH3T3 was cultured in DMEM with 5% calf serum. After passaging and culturing overnight, the medium was replaced with fresh DMEM containing different concentrations of TlNO_3_ for 24 h. The cells were harvested for further study. For the cell viability assay, 0.5 mg/mL 3-(4,5-dimethylthiazol-2-yl)-2,5-diphenyltetrazolium bromide (MTT) (Sigma) solution was added to each well of a 96-well plate and incubated for another 2 h. After aspiration, 200 µl dimethyl sulfoxide was added to each well, and the absorbance was recoded at 570 nm. For the transfection, the plasmid and TransIT-X2 (Mirus) reagent were mixed with Opti-MEM and applied to the cells. SUnSET^26^ was used to measure the global translation rate. The cells were exposed to 5 µg/mL puromycin for 10 min. The puromycin-conjugated nascent peptides were analyzed with anti-puromycin antibody (Millipore, MABE343). To detect the target proteins on the Western blots, the membranes were incubated with primary antibodies, including anti-nucleophosmin 1 (NPM1, or called B23) (Sigma, B0556), anti-fibrillarin (Abcam, Ab5821), anti-p53 (Merk, 05–224), anti-light chain 3 (LC3) B (OriGene, TA301543), anti-RpS3a (Bethyl, A305-03A), anti-RpL36 (Arigo, ARG56376), anti-tubulin (Merk, CBL270), anti-EIF2S1 (phospho S51) ((eukaryotic initiation factor (eIF) 2 alpha) (Abcam, Ab4837), anti-green fluorescent protein (GFP) (Roche, 11814460001), anti-CHOP (CST, 2895), anti-Bip (CST, 3183), and anti-histone (CST, 4499S) overnight at 4 °C, followed by a horseradish peroxidase-conjugated secondary antibody (Cell Signaling Technology). Protein signals were detected by Clarity^TM^ ECL Substrate (Bio-Rad). Images were acquired with MultiGel-21 (TopBio, Taiwan). The original pictures of Western blots are shown in the Supplemental materials.

### Northern blotting

Northern blotting was used to analyze the static state levels of pre-rRNAs. The total RNA prepared with TRI reagent (Sigma) was resolved on a formaldehyde agarose gel for large-molecular-weight rRNAs or urea polyacrylamide gel for small-molecular-weight rRNAs. The RNAs were transferred to a nitrocellulose membrane. The probes were labeled with Biotin 3ʹend labeling kit (Thermo) and continually hybridized and detected with North2South^®^ Chemiluminescent hybridization and detection kit (Thermo), respectively. Table 1 lists the probe sequences.

**Table 1:** Probes used in Northern blotting.

Probes		Sequence	Source
KLO414	5′ – ETS	CGGAGGCCCAACCTCTCCGACGACAGGTCGCCAGAGG	^51^
KLO412	ITS1	CCTCGCCCTCCGGGCTCCGGGCTCCGTTAATGATC	^51^
KLO413	ITS2	CTGCGAGGGAACCCCCAGCCGCGCA	^51^
KLO550	5′ – ITS2	GGGGCGATTGATCGGCAAGCGACGCTC	^51^
KLO551	5.8S mat	CAATGTGTCCTGCAATTCAC	^51^
KLO535	28S	TTCCTATCATTGTGAAGCAGAATTCACCAAGCGTTGGATTGTTCACCCACTAATAGGGAACGTGAGCTGGGTTTAGAC	^52^
KLO570	5S	CATCC AAGTA CTACC AGGCC C	^53^

### Sucrose gradient analysis

For the polysome analyses, the control and Tl-treated cells were treated with 0.1 mg/ml cycloheximide (CHX) for 15 min before the preparation of cell lysates (lysis buffer: 15 mM Tris-HCl (pH 7.4), 15 mM MgCl_2_, 200 mM NaCl, and 1% Triton X-100). To analyze the ratio of monosomes, the cells were harvested without CHX treatment and lysed in a low-Mg^2+^ buffer (15 mM Tris-HCl (pH 7.4), 0.25 mM MgCl_2_, 200 mM NaCl, and 1% Triton X-100). To separate the nuclear and cytoplasmic ribosomal subunits, the cells were collected from seven 10 cm dishes and lysed in a low-Mg^2+^ buffer (15 mM Tris-HCl (pH 7.4), 0.25 mM MgCl_2_, 200 mM NaCl, and 0.1% NP-40). After centrifugation at 3000 rpm, the supernatant was collected as a cytosolic fraction. The sonication buffer (25 mM Tris-HCl (pH7.5), 100 mM KCl, 2 mM ethylenediaminetetraacetic acid (EDTA), and 0.05% NP-40) was added at a 0.5 ml volume to suspend the pellet and sonicated on ice for 3 minutes. After centrifugation at 13,000 rpm for 10 min, the supernatant was collected as the nuclear fraction.

The protein extracts were loaded onto linear 10%–50% sucrose gradients in the same lysis buffer. After 2 h of centrifugation at 40,000 rpm in a SW41 Ti rotor (Beckman), the gradient fractions were collected on a density gradient fractionator (BR-188, Brandel), with continuous measurement of the absorbance at 254 nm. The fractions were collected for Western blotting. The area under the curve for each peak was calculated by Image J. The ratio of 60S to 40S or Polysome to ribosome was determined.

### Microscopy

In the immunofluorescence assays, the cells were cultured overnight in the Millicell slide (Millipore) and then exposed to different concentrations of thallium for 24 h. Cells were fixed in methanol for 20 min and acetone for 1 min. After blocking in 5% bovine serum albumin for 30 min, the cells were incubated with primary antibody diluted in a blocking solution for overnight. After washing twice with phosphate buffer saline (PBS) with Tween 20, the fluorophore-conjugated secondary antibody was added and incubated for 1 h. After washing, the samples were mounted with a mounting solution. RpL23a-GFP was amplified with forward (ATGAGATCTATGGCGCCGAAAGCGAAG) and reverse primers (GCAGAATTCTTAGATGATCCCAATTTTGTTG) and constructed in pEGFP-C1. The RpL23a-GFP was transfected with TransIT-X2 (Mirus). To detect the reactive oxygen species (ROS), the cells were stained with 2ʹ,7ʹ-dichlorofluorescin diacetate (DCFDA) (Sigma) or MitoSOX^TM^ (Thermo). Fluorescence was visualized on a microscope (AxioScope A1; Zeiss) fitted with a digital microscopy camera (AxioCam MRm Rev. 3), which was controlled with an AxioVision LE module Fluorescence Lite (Zeiss). The images were prepared using Photoshop (version CS3; Adobe).

### Quantitative polymerase chain reaction (qPCR)

The cDNAs were prepared with High-Capacity cDNA Reverse Transcription Kit (Ambion). Real-time qPCR (RT-qPCR) was performed with a Power SYBR Green PCR Master Mix (Ambion) in StepOne Real-Time PCR machine (Applied Biosystems). Table 2 lists the primer sequences.

**Table 2:** RT-qPCR primers.

Gene		Primer Sequence (F: forward, R: reverse)		Source
KLO381	RNA exonuclease 2 homolog (Rex2)	F	CAAGGCAGTGAAGGAGAGTA	This study
KLO382		R	GTTTCATGAACTGGGGCATG	
KLO392	β-actin	F	CACACTGTGCCCATCTACGA	This study
KLO393		R	GAACCGCTCATTGCCAATGG	
KLO588	p21	F	CATGTGGACCTGTCACTGTCTTGTA	(He *et al*., 2017)
KLO589		R	GAAGATCAGCCGGCGTTTG	
KLO614	c-Myc	F	AAACACAAACTTGAACAGCTAC	This study
KLO615		R	ATTTGAGGCAGTTTACATTATGG	
KLO618	NOG2	F	GATGGTCCTCAATGACTGGCAG	This study
KLO619		R	GGACAACTTCCAAAGATGAGGAG	
KLO622	NOG1	F	CCGTTTGCCAACCATTGATCCG	This study
KLO623		R	GTTGTGAACGCATAGGGCTGGA	

### Flow cytometry

To analyze the status of the cell cycle, the cells treated with Tl(I) for 24 h were collected in the tubes. The cells were fixed in cold PBS and methanol solution for 2 h at 4 °C. After washing with PBS, the cells were incubated with 550 U/mL RNase and 50 µg/ml propidium iodide (PI) for 30 min at 37 °C. To analyze the fraction of apoptotic cells, the cells were labeled with Annexin V–FITC apoptosis detection kit (Strong Biotech Co.). Approximately 10,000/sample cells were analyzed using a flow cytometer (FC500, Beckman Coulter, USA).

### Next-generation sequencing (NGS) Analysis

The RNA was prepared in duplicate from cells treated with Tl(I) for 24 h using RNAzol (Sigma). After the RNA samples passed the quality controls, the mRNAs were purified with oligo(dT) beads. The mRNAs were then fragmented randomly, followed by cDNA synthesis using hexamers and the reverse transcriptase. The sequences were analyzed with HiSeq. 4000 (Illumina). The mRNA expression was analyzed with TopHat2. The gene expression values were calculated as fragments per kilo base of exon per million fragments mapped (FPKM), and HTSeq software was used to analyze the gene expression levels in the experiments. The Kyoto Encyclopedia of Genes and Genomes (KEGG) was utilized to generate the enrichment list and scattered plots.

## Supplementary information

Supplementary
