# Supplementary material for: Thallium(I) treatment induces nucleolar stress to stop protein synthesis and cell growth
Source: Sci Rep. 2019 May 6;9:6905. doi: 10.1038/s41598-019-43413-1 (PMC6502789; doi:10.1038/s41598-019-43413-1)

Online Supplemental File

Thallium(I) treatment induces nucleolar stress to stop protein synthesis and cell growth

Yi-Ting Chou, Kai-Yin Lo*

Department of Agricultural Chemistry, National Taiwan University, Taipei, Taiwan

*corresponding author:

Kai-Yin Lo, PhD
Department of Agricultural Chemistry
College of Bioresources and Agriculture
National Taiwan University
Taipei 10617, Taiwan

TEL: 886-2-33669440
email: kaiyin@ntu.edu.tw

Fig 3A

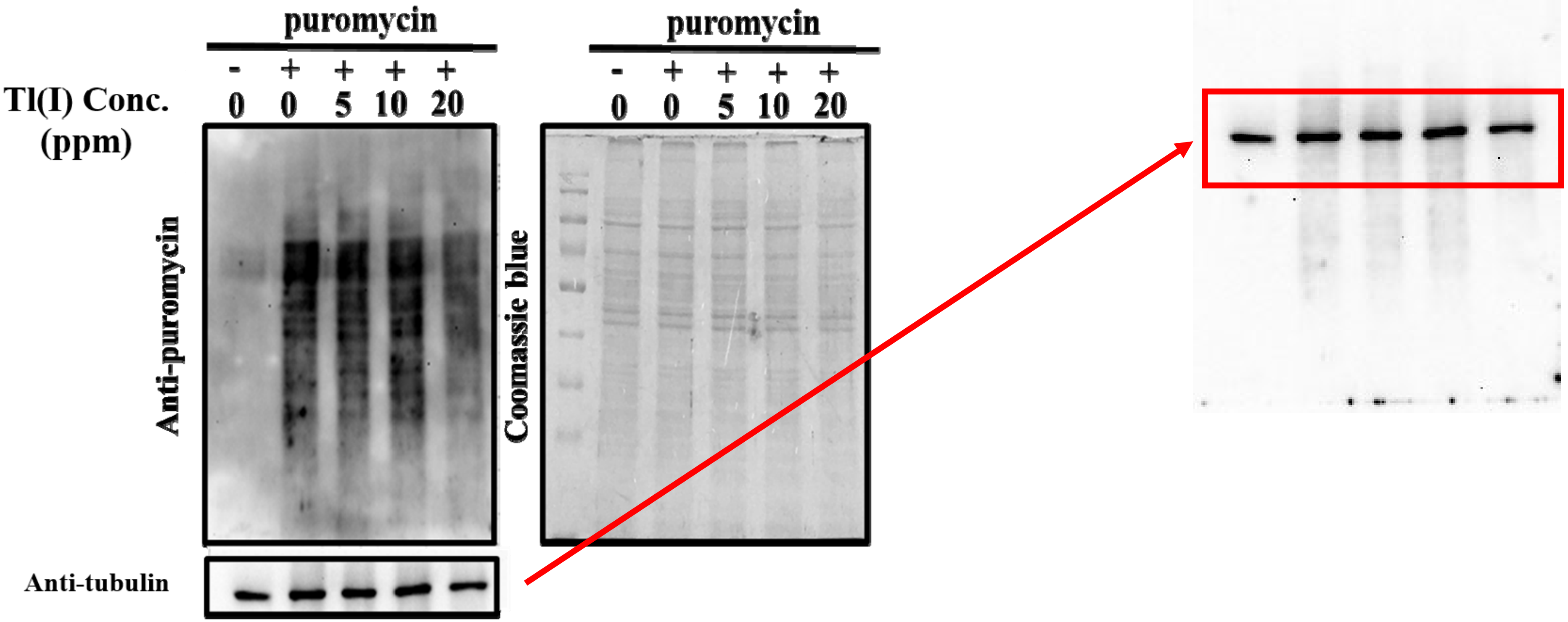
puromycin
puromycin
Tl(I) Conc. (ppm)
- + + + +
0 0 5 10 20
Anti-puromycin
Coomassie blue
Anti-tubulin

Fig 3D

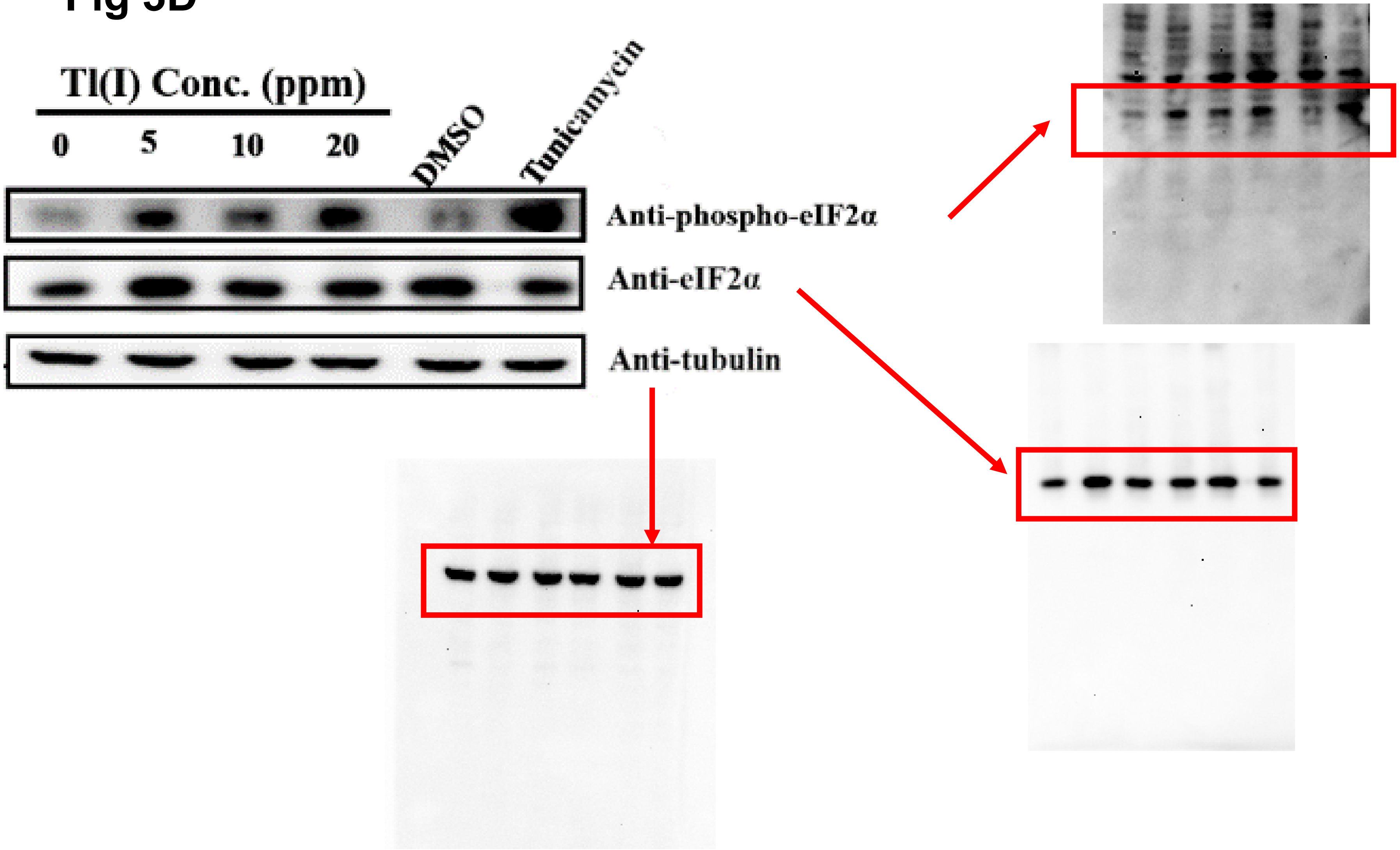
Tl(I) Conc. (ppm)
0
5
10
20
DMSO
Tunicamycin
Anti-phospho-eIF2α
Anti-eIF2α
Anti-tubulin

Fig 4C

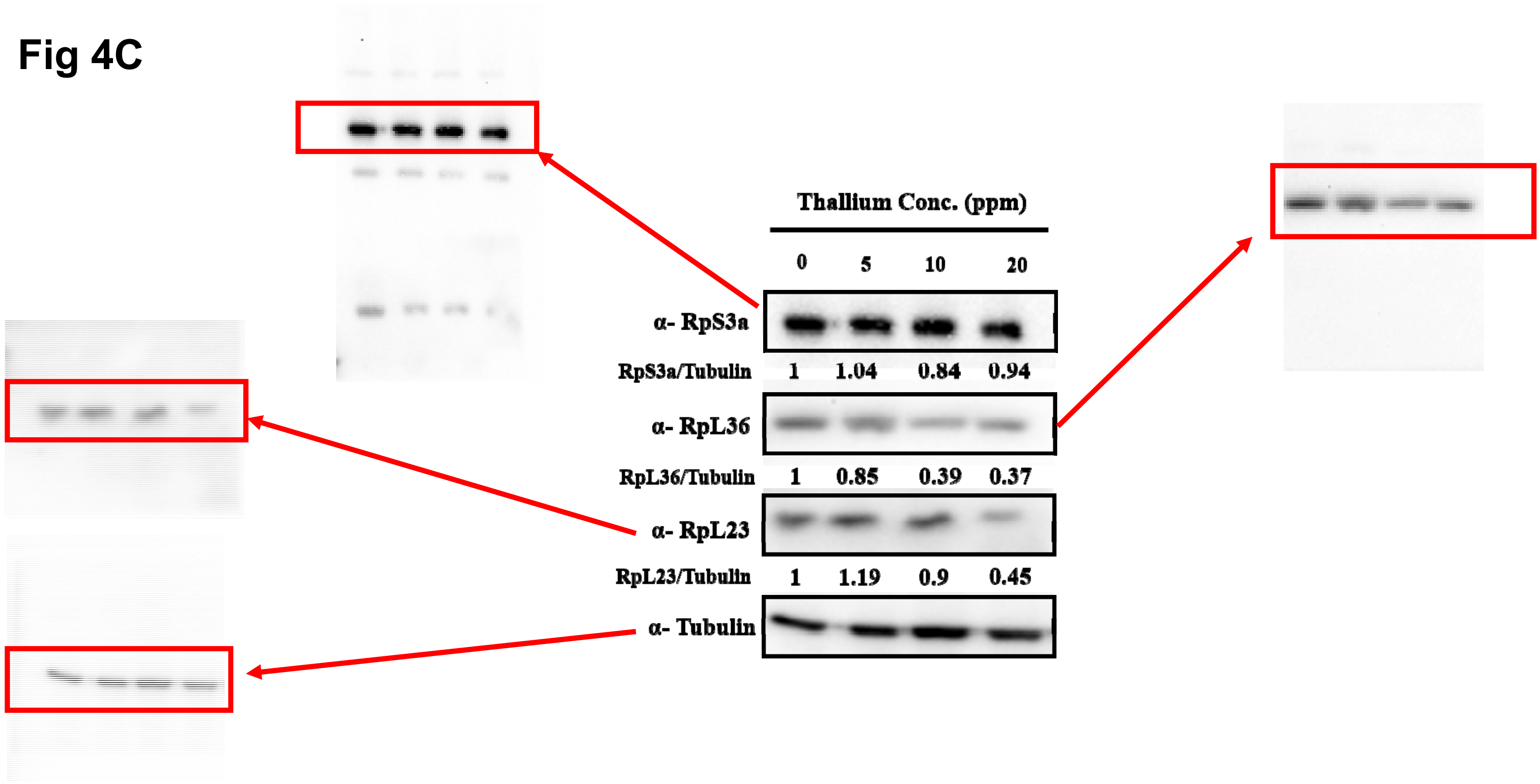

Thallium Conc. (ppm)
0
5
10
20
α- RpS3a
RpS3a/Tubulin
1
1.04
0.84
0.94
α- RpL36
RpL36/Tubulin
1
0.85
0.39
0.37
α- RpL23
RpL23/Tubulin
1
1.19
0.9
0.45
α- Tubulin

Fig 5A

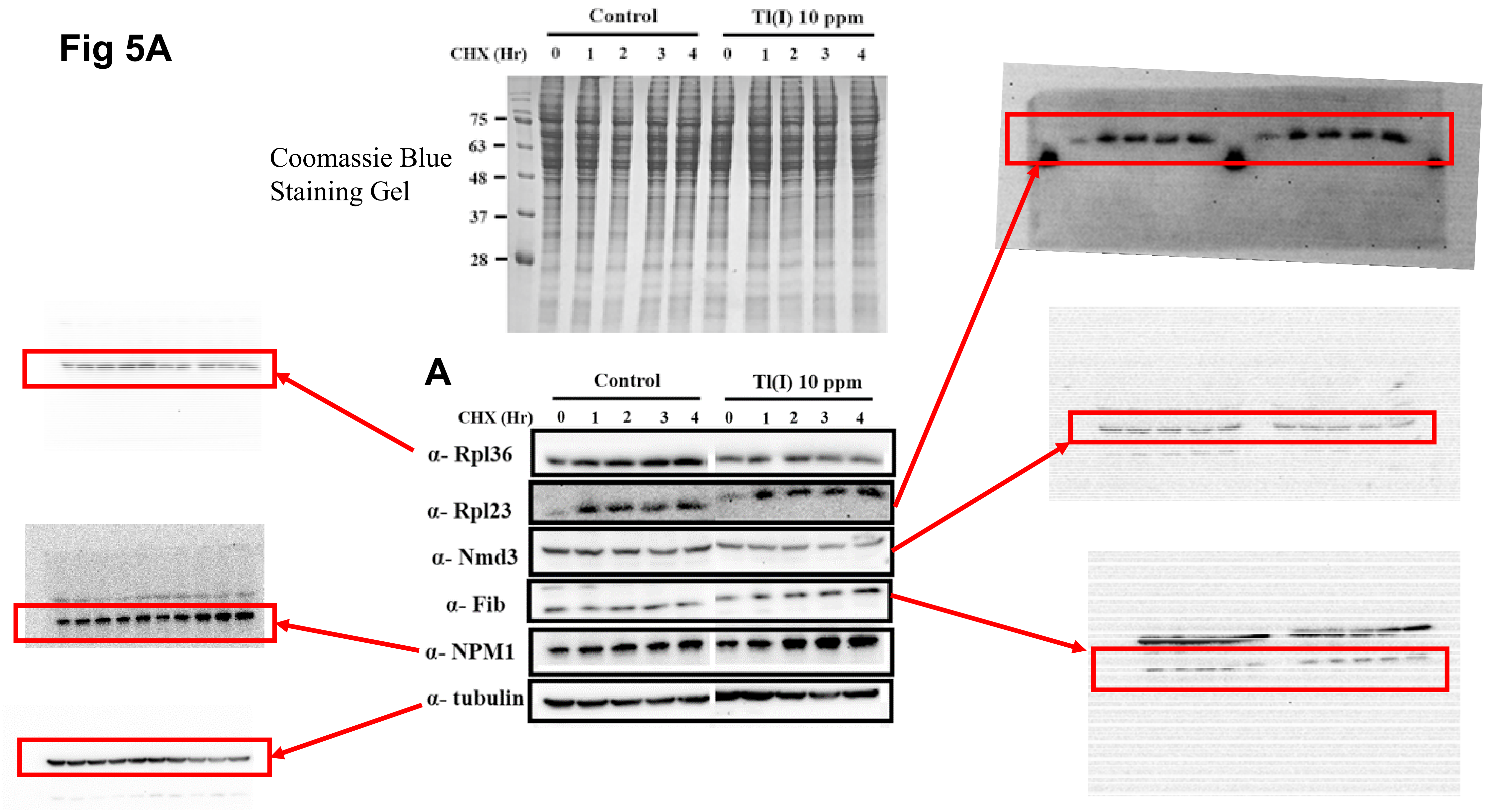

Coomassie Blue
Staining Gel
Control
Tl(I) 10 ppm
CHX (Hr)
0 1 2 3 4 0 1 2 3 4
75
63
48
37
28
A
α- Rpl36
α- Rpl23
α- Nmd3
α- Fib
α- NPM1
α- tubulin

Fig 5B

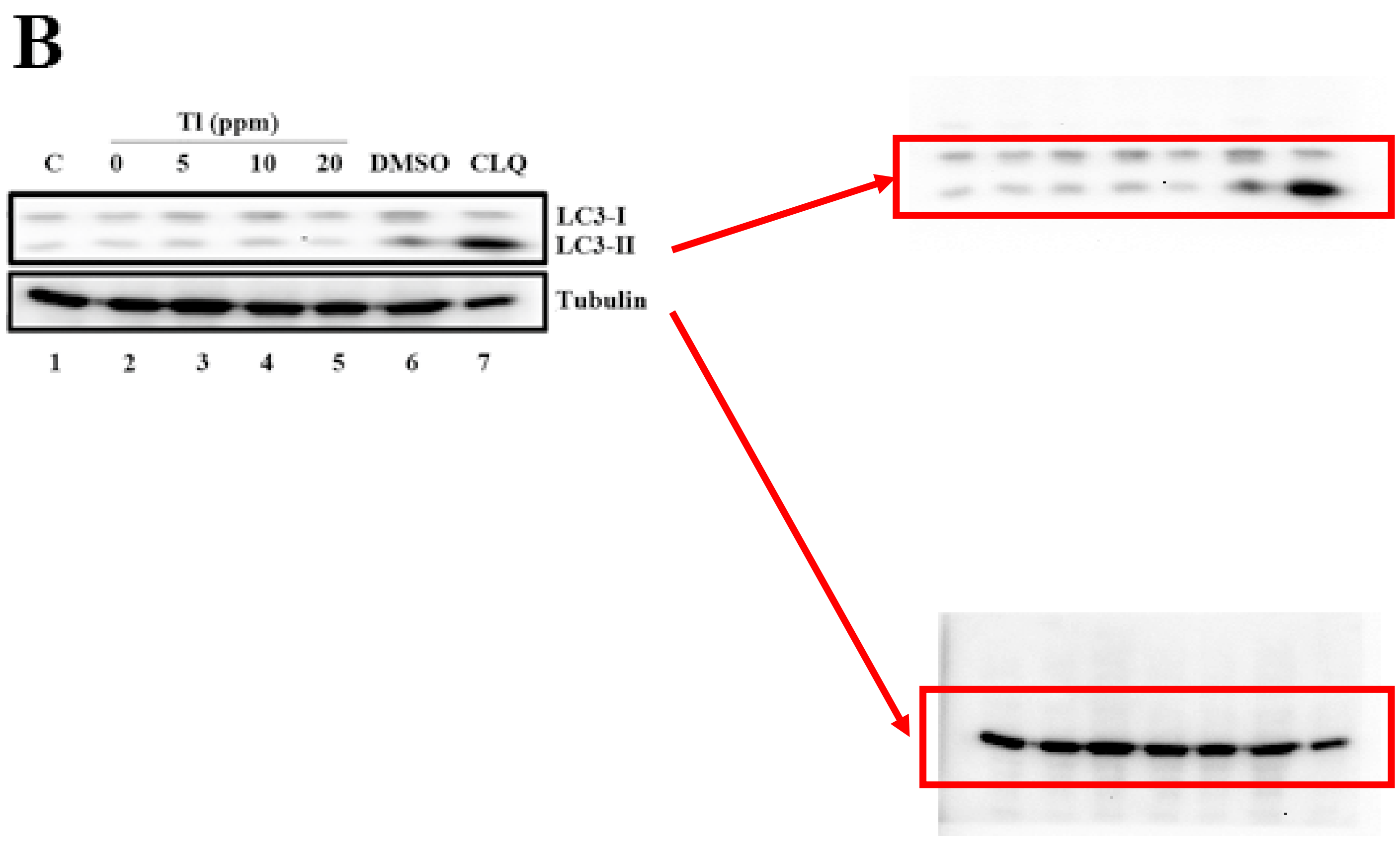

Fig 5C

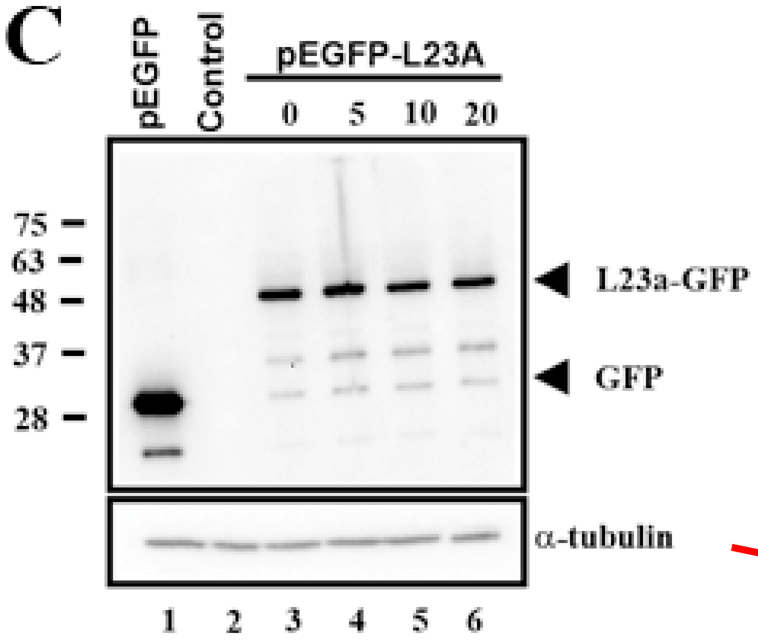
C
pEGFP
Control
pEGFP-L23A
0
5
10
20
75
63
48
37
28
L23a-GFP
GFP
α-tubulin
1
2
3
4
5
6

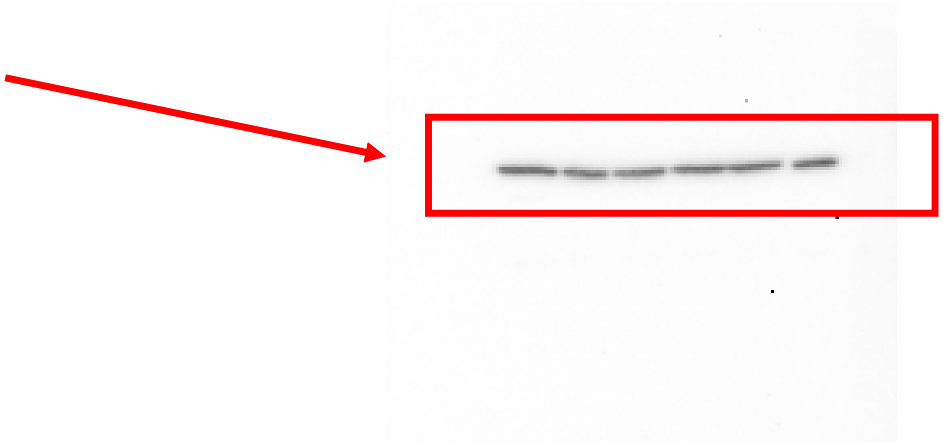

Fig 6B

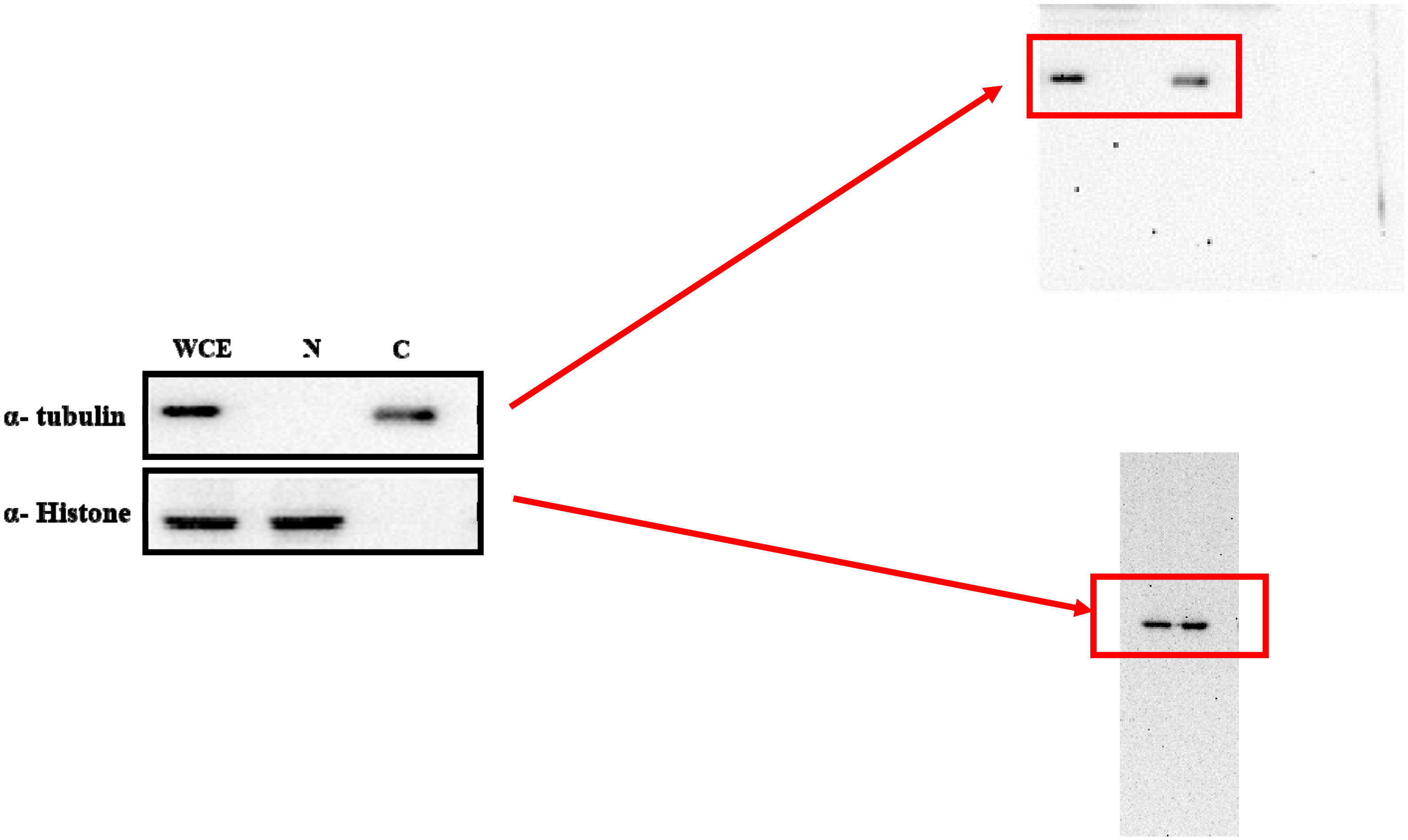

Fig 7 S1

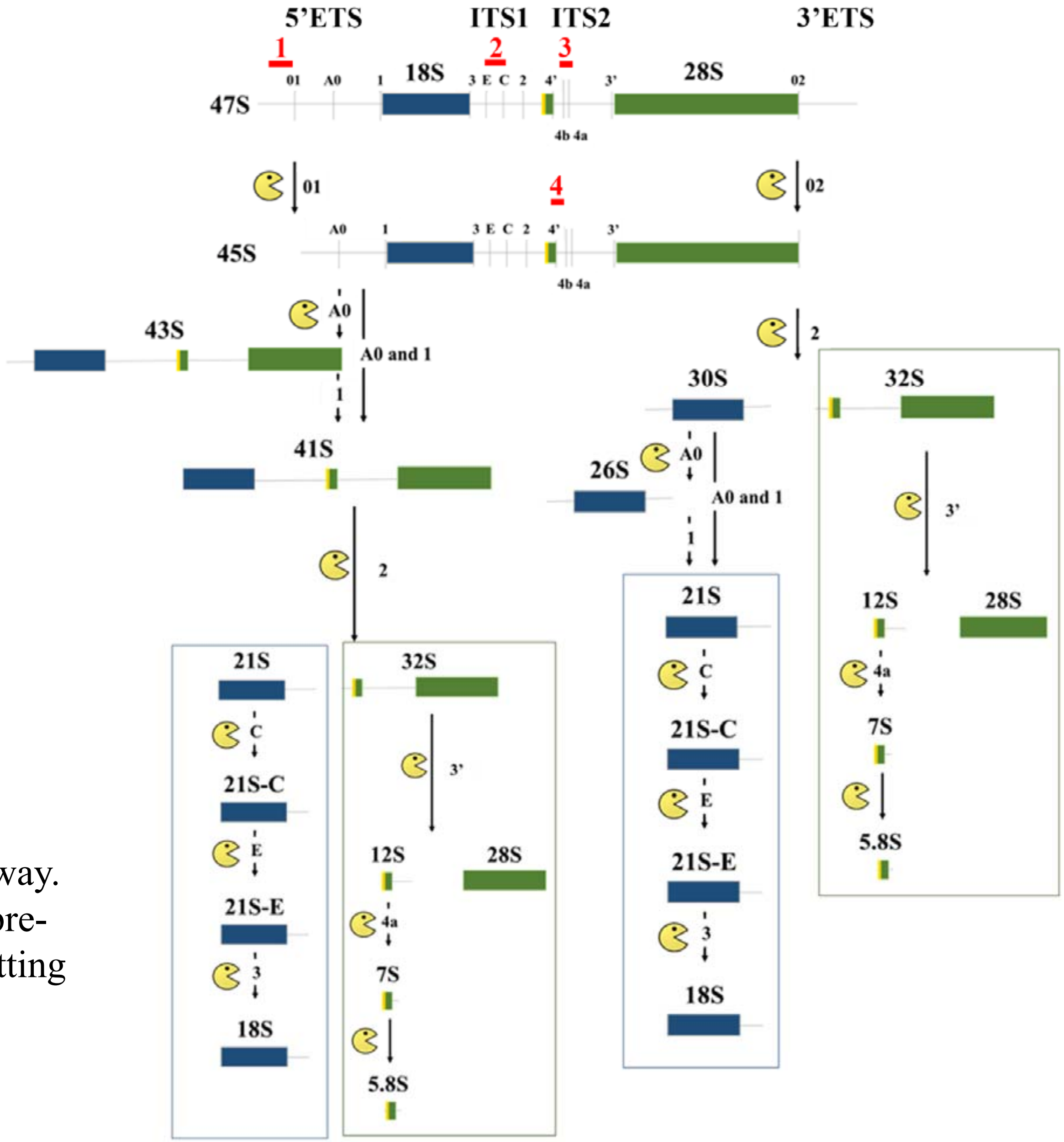

Pre-rRNA processing pathway. The probes used to detect pre-rRNAs in the Northern blotting were shown in red bars.

Fig 7D

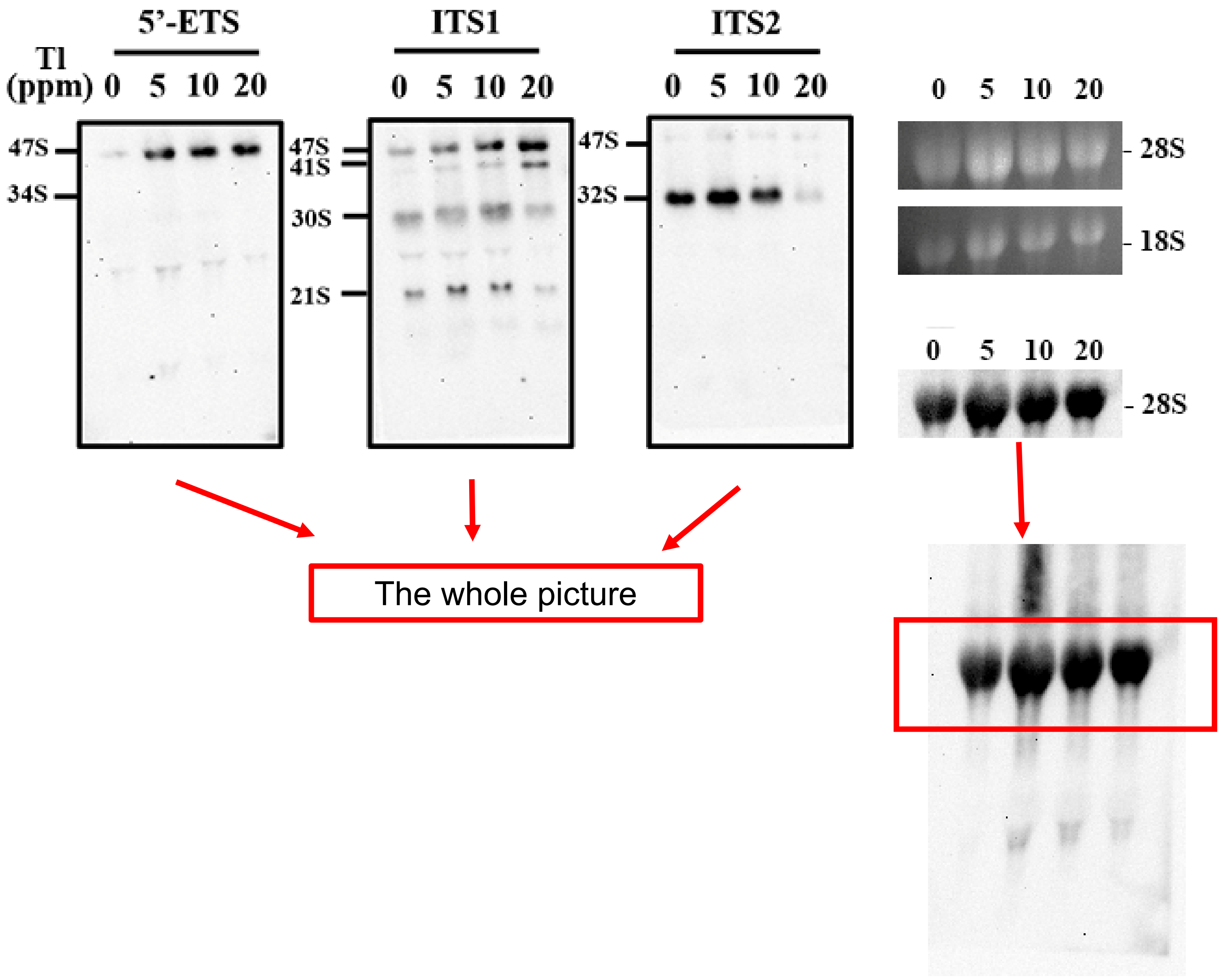
5'-ETS
ITS1
ITS2
Tl (ppm) 0 5 10 20
0 5 10 20
0 5 10 20
0 5 10 20
47S
34S
47S
41S
30S
21S
47S
32S
28S
18S
0 5 10 20
28S
The whole picture

Fig 7E

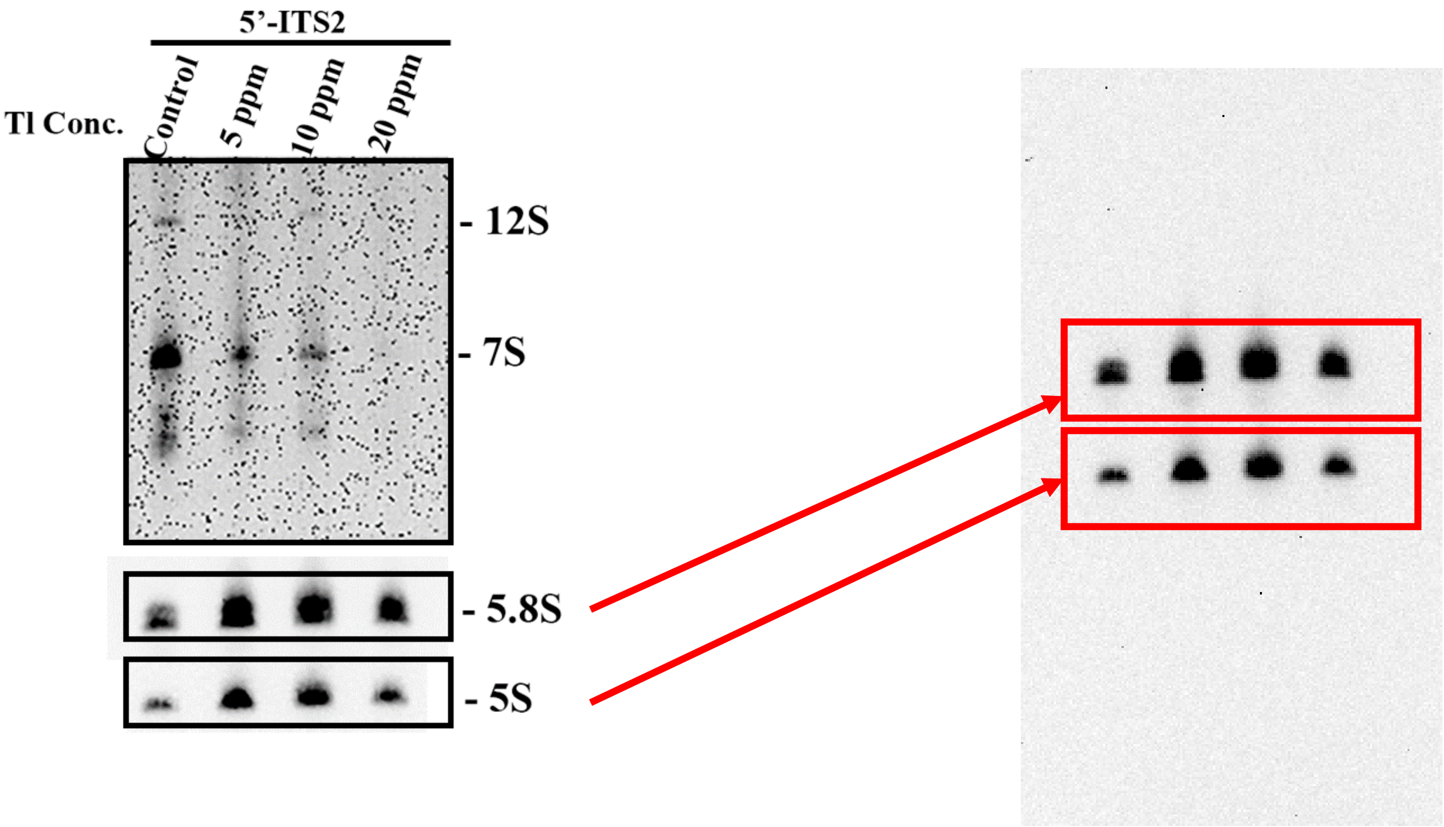

Fig 9D

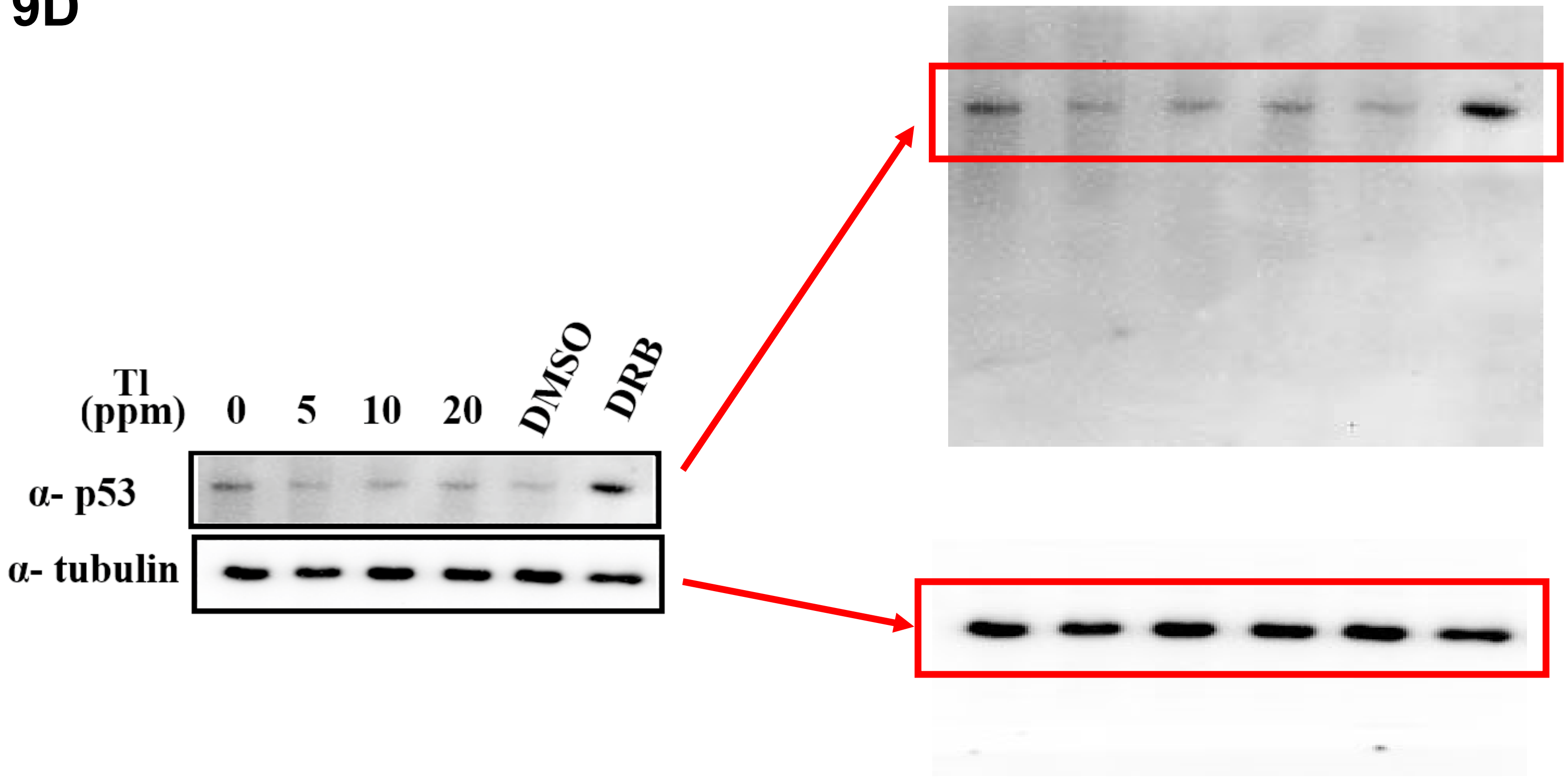

Supplement: Supplementary file 1 — Supplementary [file 41598_2019_43413_MOESM1_ESM.pdf]
